# Naltrexone Has Variable and Schedule-Dependent Effects on Oral Squamous Cell Carcinoma Cells

**DOI:** 10.3390/ijms262110651

**Published:** 2025-11-01

**Authors:** Sahar Kazmi, Erica Sanford, Zaid A. Rammaha, Ethan J. Bengson, Feng Gao, Linda Sangalli, Cai M. Roberts

**Affiliations:** 1 College of Dental Medicine—Illinois, Midwestern University, Downers Grove, IL 60515, USA; sahar.kazmi@midwestern.edu (S.K.); erica.sanford@midwestern.edu (E.S.); fgao@midwestern.edu (F.G.); 2 Biomedical Sciences Program, Midwestern University, Downers Grove, IL 60515, USA; zaid.a.ramaha@gmail.com (Z.A.R.); ethan.bengson@midwestern.edu (E.J.B.); 3 Department of Pharmacology, Midwestern University, Downers Grove, IL 60515, USA

**Keywords:** oral squamous cell carcinoma, naltrexone, opioid growth factor receptor, chemotherapy

## Abstract

Oral squamous cell carcinoma (OSCC) is marked by profound differences in survival between the localized and disseminated disease, estimated to result in a 70% and less than a 40% five-year survival rate with surgical and/or radiation approaches (in localized cases) and chemotherapy (in metastatic cases), respectively. Given the suboptimal efficacy of current management options, new therapeutic approaches are needed to supplement existing chemotherapies and improve outcomes. One emerging therapeutic option is naltrexone (NTX), an opioid antagonist that has shown promising outcomes at low doses in other forms of cancer. This study sought to determine the effectiveness of intermittent dosing of naltrexone on oral cancer cell survival, either as a single agent or in combination with traditional chemotherapy. Two human OSCC lines (locally invasive SCC-25 and metastatic Detroit 562) were cultured. Cells were exposed to 1 µM and 10 µM NTX alone, using intermittent (5 h once, 5 h daily, 5 h every other day) or constant 72 h exposure. Cells were exposed to combination therapy with cisplatin or docetaxel under three NTX regimens (5 h, 24 h, and continuous). Cell viability was determined using Sulphorhodamine B (SRB) assay and Cell Counting Kit-8 (CCK-8). Differences across treatments were assessed using ANOVA (*p* < 0.05). The effect of low-dose NTX alone, across varying treatment regimens, did not yield significant, consistent changes in OSCC cell survival. Combination with cytotoxic drugs reduced cell viability more efficiently than chemotherapy alone at select doses, particularly through intermittent short-term pretreatment schedules, but the full dose response demonstrated antagonism between NTX and chemotherapy, independent of the dosing schedule. These results contrast with previous findings in other cancers, and, thus, further study and optimization will be needed to determine the clinical benefit and reproducibility of these findings.

## 1. Introduction

Oral squamous cell carcinoma (OSCC) is a major public health burden and the most common malignancy affecting the oral cavity [1]. It ranks among the top 20 most common cancers worldwide and accounts for approximately 390,000 of new cases globally [2]. The disease trajectory and survival outcome vary profoundly depending on the stage of diagnosis. When OSCC is detected early and remains confined to the primary site, standard treatment typically consists of surgical resection with or without adjuvant radiation [3], resulting in five-year survival rates of about 70% [4]. In contrast, metastatic OSCC often necessitates systemic chemotherapy, where treatment efficacy is hindered by drug resistance, side effects, and limited durability of the response, contributing to a five-year survival rate of less than 40% [5,6,7,8,9]. Alarmingly, 60% of OSCC cases are identified at an advanced stage (stage III or higher), dictating a much poorer prognosis [10]. Despite advances in surgical techniques, radiation protocols, and chemotherapeutic agents, the overall five-year survival rate has plateaued at around 50% for the past two decades [11]. Furthermore, projections from the Global Cancer Observatory indicate that by 2040, the incidence rate of OSCC will rise by approximately 40%, and its associated mortality will increase by 30%, underscoring the urgency for improved therapeutic approaches [12,13].

Recent studies have investigated novel agents to supplement traditional management options. Naltrexone (NTX) is a non-selective competitive opioid receptor antagonist approved by the Food and Drug Administration (FDA) for addiction treatment at dosages between 50 and 150 mg. At low doses (1–5 mg), NTX has been used off-label to treat chronic pain and various systemic inflammatory and autoimmune conditions [14,15,16,17,18,19,20]. In addition to its pain modulation, early evidence has suggested that low-dose NTX may be a promising anticancer treatment option [19,21]. One proposed mechanism of action for its efficacy in cancer therapy involves the blockage of the opioid growth factor ([Met^5^]-enkephalin, OGF) and its receptor (OGFr), a regulatory axis suggested to inhibit cell proliferation in human cancer and normal cells [22,23] through the modulation of the G1/S cell cycle phase via cyclin-dependent kinase inhibitory pathways [24,25,26]. Some evidence suggests that the OGFr may be markedly reduced in human head and neck squamous cell carcinoma, with tumor tissue exhibiting ninefold fewer OGFr binding sites and a fivefold reduction in OGFr protein levels compared to controls [27], thus claiming a dysregulation of this modulatory pathway in cancer disease progression [23,28]. While in vitro and animal studies have suggested that NTX may restore the functionality of the OGF-OGFr axis and suppress tumor growth [25,29,30], the mechanistic OGF-OGFr effect has not been consistently replicated across the literature and findings remain controversial regarding dosing, treatment schedule, and mechanism of action [21]. 

To address these gaps, the primary aim of this study was to investigate the efficacy of various doses, durations, and schedules of NTX on the viability of OSCC, in comparison to control cells and variations in OGF-OFGr expression as a function of the tumor stage and NTX treatment. Based on the previous literature [31,32], we hypothesized that the treatment of OSCC with the intermittent administration of NTX would inhibit OSCC cell growth. We also hypothesized that OGF-OGFr expression would differ between OSCC and control cells, and would be modulated as a result of NTX therapy. We also hypothesized that NTX may sensitize OSCC cells to other therapies. Thus, a secondary aim was to analyze the efficacy of NTX in combination with standard cytotoxic chemotherapeutic agents and its effect on short-term changes in OGFr expression in treated cells. Finally, to test for differences in NTX response between tumor stages, we chose two cell line models representing locally invasive and metastatic carcinoma: SCC-25 and Detroit 562, respectively. We have previously shown that these lines differ in their biomarker expression and drug response, supporting their use in the present study [33].

## 2. Results

### 2.1. OSCC Cells Express OGFR

We selected the lines SCC-25 and Detroit 562 in order to test cells from both locally invasive and metastatic cells. Prior work has shown that head and neck squamous carcinoma cells have similar gene expression profiles regardless of their tissue of origin, thus we consider these to be adequate models of squamous carcinoma progression [34]. First, we verified that OGFrs were expressed in our lines by comparing them to normal human Primary Gingival Keratinocytes (PGK) cells, and saw that SCC-25 had a similar OGFr protein expression, while Detroit 562 cells had somewhat higher levels of OGFrs (*n* = 1, Supplementary Figure S1a). This could be because the Detroit 562 cell line is a pharyngeal carcinoma line derived from metastatic tissue, which has increased proliferation and altered regulatory pathways, causing dysregulation.

### 2.2. Low-Dose Naltrexone Does Not Significantly Impact OSCC Cell Survival

**Figure 1:**
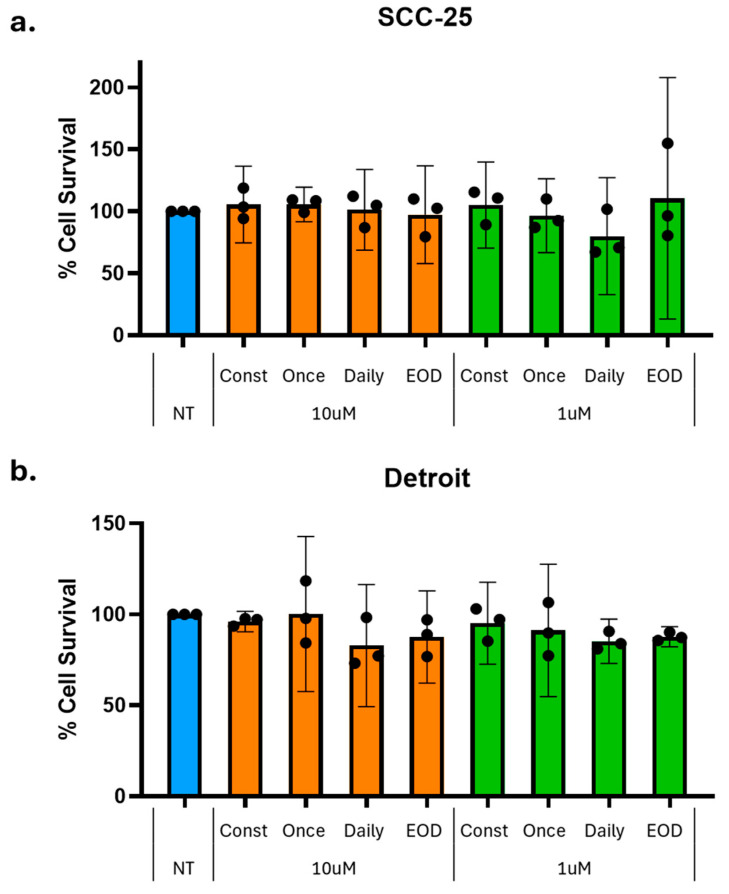
Evaluation of NTX efficacy in cell lines. Two doses were tested in (**a**) SCC-25 and (**b**) Detroit 562 cells. Each dose was tested with constant 72 h exposure (Const), single 5 h treatment (Once), 5 h treatment daily for three days (Daily), or 5 h treatment every other day (EOD). NT, no treatment control (blue); 10 µM dose (orange); 1 µM dose (green). Any inhibition of growth was not repeatable, and no statistically significant changes in cell survival were observed (one-way ANOVA, SCC-25 *n* = 4, Detroit 562 *n* = 3, bars represent mean ± 95% confidence interval (CI)). Dots represent individual values.

To evaluate the efficacy of NTX, two different doses were tested in two distinct head and neck cancer cell lines: SCC-25 and Detroit 562. Each NTX dose was used in a schedule comprising constant 72 h exposure (Const), single 5 h treatment (Once), 5 h treatment daily for three days (Daily), or 5 h treatment every other day (EOD). Any inhibition of growth was not repeatable, and no statistically significant changes in cell survival were observed in either SCC-25 (*n* = 4, Figure 1a) or Detroit 562 (*n* = 3, Figure 1b).

### 2.3. Naltrexone Has Mild Effects on SKOV-3 Ovarian Cancer Cells

With the previous literature proving that NTX affects the growth of SKOV-3 ovarian cancer cells [29], we repeated our experiments in this cell line to assess the efficacy of our dosage and timing schedules of NTX treatment. We first verified that the OGFr expression was similar in SKOV-3 to what we had observed in SCC-25 and Detroit 562 (*n* = 1, Supplementary Figure S1b). When SKOV-3 was plated at low density, using an equal starting cell number to the OSCC cells, constant NTX drove cell growth, while intermittent dosing (i.e., once, daily, or EOD) had a slight suppressive effect, up to 10µM, though these changes were not statistically significant (Supplementary Figure S2a). At higher doses, all dosing schemes were toxic (*n* = 1, Supplementary Figure S2b). However, we noted SKOV-3 cells grew more slowly than the OSCC lines, which may have affected our ability to detect significant changes. We therefore repeated the experiment with 3000 cells plated per well, producing an ending density similar to the OSCC lines. Variability increased, and no statistically significant changes were observed, though some replicates led to reduced growth in the presence of intermittent NTX, particularly EOD (Supplementary Figure S2c). While not completely transparent results, these findings do suggest that the efficacy and cellular response to NTX in SKOV-3 cells and OSCC cells are similar and may be influenced by both dosing strategy and cell density, highlighting the importance of validating methods when evaluating NTX treatment outcomes.

### 2.4. Select Doses of Naltrexone Enhance the Efficacy of Chemotherapy in OSCC Cells

**Figure 2:**
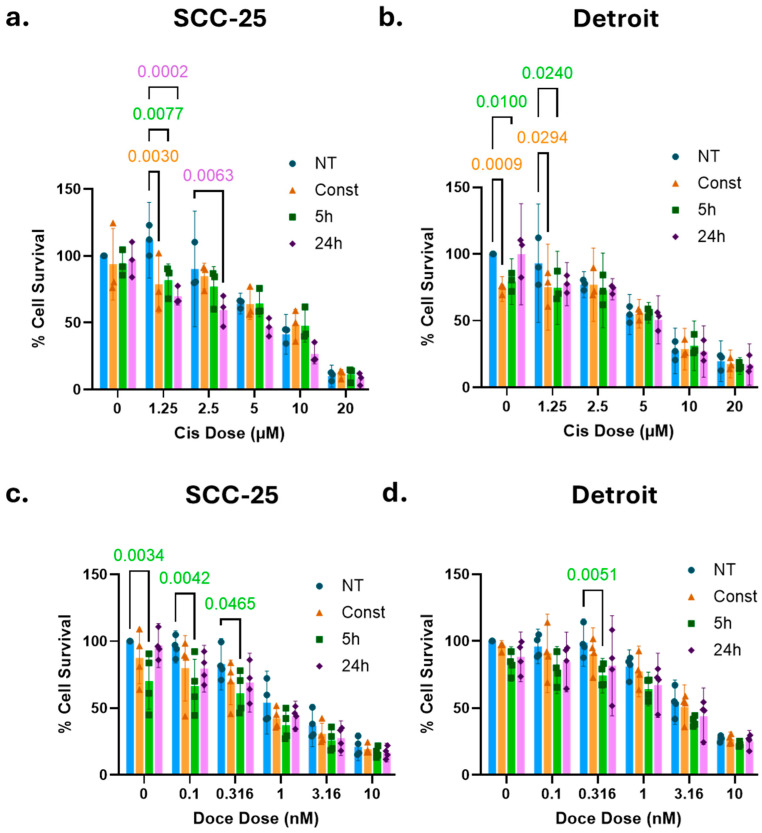
Effects of combining NTX with chemotherapy. (**a**) In SCC-25, NTX sensitized cells to cisplatin (Cis) vs. no NTX treatment (NT), with 24 h exposure performing the best. (**b**) Combination of NTX with Cis in Detroit 562 cells. Constant and 5 h exposure to NTX had a slight sensitizing effect. (**c**,**d**) Combination of NTX with docetaxel (Doce) was more effective than Doce alone in both SCC-25 (**c**) and Detroit 562 (**d**) cells. The 5 h exposure time to NTX had a statistically significant effect in both lines. Data were analyzed using two-way ANOVA, with Dunnett’s test comparing conditions at each Cis/Doce dose. Graphs show mean ± 95% CI of *n* = 3 (Cis) or *n* = 4 (Doce) independent experiments. Exact *p* values compared to NT are shown with text colored the same as the corresponding condition. Blue, NT; orange, constant; green, 5 h exposure; purple, 24 h exposure.

Current chemotherapeutic regimens for OSCC consist of platinum, taxane, and/or fluorouracil. Recent trials have used all three drugs in combination [35,36,37]. In order to survey chemotherapy drugs with different mechanisms of action (i.e., DNA damage and spindle poison), we selected cisplatin and docetaxel, two agents with which our laboratory is familiar [33,38]. To evaluate the potential of naltrexone (NTX) as an adjunct to standard chemotherapy, SCC-25 and Detroit 562 cells were treated with cisplatin or docetaxel in combination with 10 µM NTX under three exposure schedules: 5 h pretreatment followed by 19 h normal media, 24 h pretreatment, and continuous treatment (24 h pretreatment plus continued NTX presence during chemotherapy exposure). After the initial 24 h pretreatment window, cells were treated with increasing concentrations of either cisplatin or docetaxel, and viability was assessed after 48 h. Across both cell lines, the NTX treatment resulted in decreased cell survival compared to chemotherapy alone, with short-term exposures (5 h and 24 h) generally producing stronger effects than continuous treatment (Figure 2). The degree of sensitization varied depending on both the chemotherapy agent used and the NTX exposure. NTX sensitized SCC-25 cells to cisplatin, particularly when the 24 h pretreatment was used (Figure 2a). In Detroit 562 cells, significant changes from the no-treatment controls were only observed with low or absent levels of cisplatin (Figure 2b). In both cell lines, the treatment with 5 h of NTX prior to docetaxel was more efficacious than docetaxel alone (Figure 2c,d). These results suggest that NTX may increase the cytotoxic effect of chemotherapeutic agents in a cell-line-dependent and specific manner. Shorter NTX exposures appeared to be more effective than continuous treatment, indicating that timing plays a critical role in maximizing the benefit. Interestingly, in two combinations, NTX alone had a statistically significant effect on viability (Figure 2b,c). This may be due to the lower variability or increased sensitivity of CCK-8 assays. Together, these data suggest that 10 µM NTX could be a chemosensitizing agent in OSCC therapy.

### 2.5. Naltrexone and Chemotherapy Do Not Show Consistent Synergistic Effects

**Figure 3:**
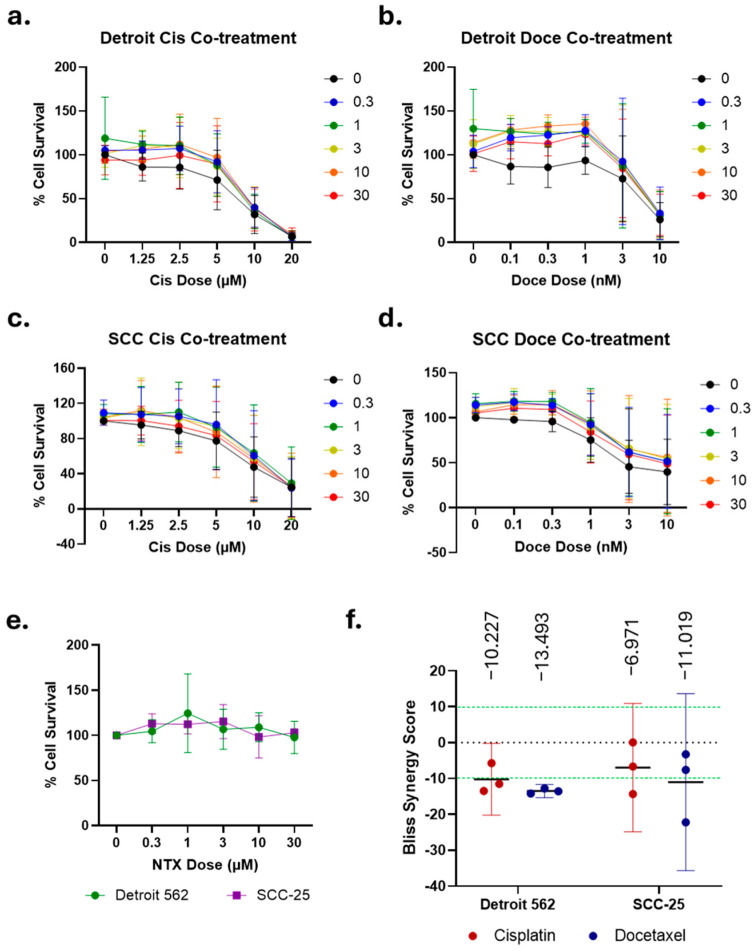
Dose responses and synergy analyses for NTX and chemotherapy co-treatment. Detroit 562 and SCC-25 cells were treated with the indicated doses of cisplatin (**a**,**c**) or docetaxel (**b**,**d**). NTX doses in µM are indicated by line color. Mean and SD of three independent experiments shown for each plot. (**e**) Pooled NTX-only data from experiments in (**a**–**d**) demonstrate no cytotoxic effect in the absence of chemotherapy. Mean ± 95% CI is shown. (**f**) Bliss synergy scores for the indicated drug combinations. Mean, 95% CI, and individual points from each experiment are shown. Average Bliss score appears above the graph.

**Figure 4:**
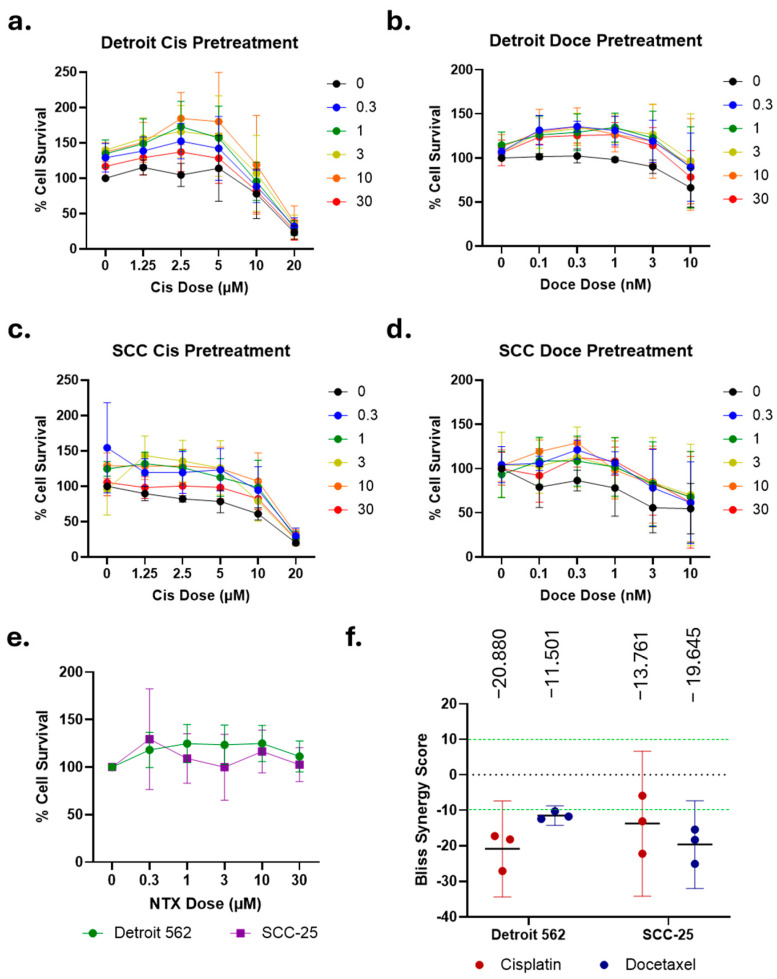
Dose responses and synergy analyses for chemotherapy after 5 h pretreatment with NTX. Detroit 562 and SCC-25 cells were treated with the indicated doses of cisplatin (**a**,**c**) or docetaxel (**b**,**d**). NTX doses in µM are indicated by line color. Mean and SD of three independent experiments shown for each plot. (**e**) Pooled NTX-only data from experiments in (**a**–**d**) demonstrate no cytotoxic effect in the absence of chemotherapy. Mean ± 95% CI is shown. (**f**) Bliss synergy scores for the indicated drug combinations. Mean, 95% CI, and individual points from each experiment are shown. Average Bliss score appears above the graph.

**Figure 5:**
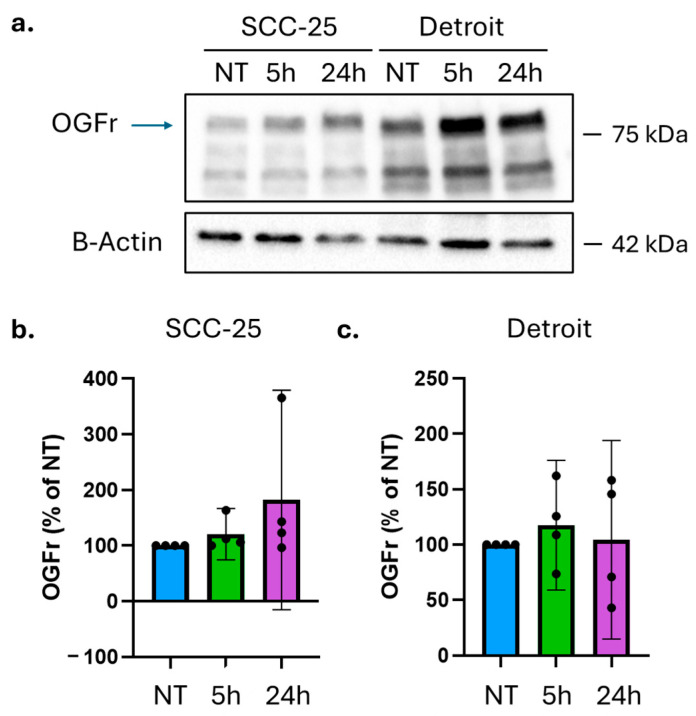
Evaluation of OGFr expression in cell lines. (**a**) OGFr expression was compared in untreated SCC-25 cells (NT), those treated with 10µM NTX for five hours (5 h), and those treated for twenty-four hours (24 h). Representative blot shown. (**b**) Quantification of *n* = 4 replicate experiments shows a trend toward increased OGFr expression in NTX-treated SCC-25 cells that did not achieve statistical significance due to high variability. (**c**) Detroit 562 cells treated with NTX do not show a consistent trend in OGFr expression changes (*n* = 4). Graphs show mean ± 95% CI. Dots represent individual values. OGFr observed molecular weight, 90 kDa (arrow). Blue, NT; green, 5 h treatment; purple, 24 h treatment.

In order to determine if NTX and the selected chemotherapeutics are truly more efficacious in combination, we next performed assays with a range of doses for both NTX and either cisplatin or docetaxel for dose response and synergy analyses. Surprisingly, neither concurrent NTX exposure (Figure 3a–d) nor 5 h NTX pretreatment (Figure 4a–d) significantly altered the response to chemotherapy. The IC_50_ values for cisplatin remained approximately 10 µM for both cell lines with NTX co-treatment, and were slightly higher for those with NTX pretreatment (Figure 3a,c and Figure 4a,c). The IC_50_ values for docetaxel ranged from 3 to 10 nM in co-treatment experiments, and the IC_50_ was not reached for pretreatment with docetaxel, most likely due to the shorter exposure time in these assays (Figure 3b,d and Figure 4b,d). Analyses of all the NTX-only data, from all conditions, were pooled to derive a dose response for NTX. Neither 72 h (co-treatment, Figure 3e) or 5 h of exposure to NTX (pretreatment, Figure 4e) led to any appreciable cell death, reaffirming the lack of effect seen with limited doses and additional timings (Figure 1). Finally, we used SynergyFinder to analyze these assays for synergy via the Bliss Independence score, selected due to the agents functioning in different cellular pathways. Bliss score values > 10 indicate synergy, scores ~0 indicate additivity, and scores <−10 indicate antagonism. NTX co-treatment led to average scores ranging from −13.493 to −6.971, indicating weak antagonism (Figure 3f). NTX pretreatment produced average scores from −20.880 to −11.501, suggesting slightly stronger antagonism (Figure 4f). Representative synergy plots from each condition are given in Supplementary Figures S3 and S4. These data agree with prior cell line studies demonstrating that continuous exposure to NTX increases viability, which would naturally oppose the action of chemotherapy [21]. However, it is interesting that intermittent dosing here produces the same result.

### 2.6. Effect of Naltrexone Exposure on OGFr Expression Is Minimal and Cell-Line-Dependent

OGFr expressions were compared in untreated OSCC cells, those treated with 10 µM NTX once for 5 h followed by 19 h of normal media, and those treated with constant NTX exposure for the full 24 h. In SCC-25 cells, there was a trend toward an increased OGFr expression with NTX treatment duration, but due to inter-experiment variation, the difference did not achieve statistical significance (Figure 5a,b, *n* = 4). No clear trend was observed in OGFr expression following NTX exposure in Detroit 562 cells (Figure 5a,c, *n* = 4). Therefore, it is unlikely that NTX treatment under these conditions significantly alters OGFr expressions, which may partially explain the lack of efficacy in both single-agent and full combination studies.

## 3. Discussion

OSCC remains a major clinical challenge, particularly when diagnosed at an advanced stage, where treatment options are limited and outcomes are often poor. Thus, there is a growing need for adjuvant therapies that can enhance efficacy without introducing substantial toxicity. In this study, we evaluated the effect of NTX, alone and in combination with existing chemotherapeutic agents, on OSCC cell viability and OFGr expression. We selected two cell lines representing different stages of the disease progression at distinct anatomical sites and evaluated their responses to different NTX exposure regimens.

### 3.1. Minimal Effect of NTX on OSCC Cell Survival

As a monotherapy, NTX demonstrated minimal impact on OSCC cell viability, with no consistent or reproducible inhibition of cell proliferation observed across the experiment replicates or cell lines (SCC-25 or Detroit 562), regardless of the NTX exposure regimen (Figure 1). Similarly to our findings, a systematic review [21] highlighted the heterogeneity of NTX exposure regimens across in vitro cancer cell studies where NTX has been administered intermittently, continuously, or for a short-term duration, for between 72 and 120 h, at concentrations between 10^−6^ and 10^−5^ M. In the current study, we implemented several exposure schedules (continuous treatment for 72 h, a single 5 h exposure, repeated daily 5 h exposure over three days, or 5 h exposure every other day), informed by previous studies suggesting that intermittent or short-term NTX may be more effective than continuous treatment in inhibiting tumor growth [21,39]. Indeed, previous studies have shown that short-term NTX exposure can inhibit tumor cell growth by 24% to 42% across multiple cancer cell lines [21], including SKOV-3 and OVCAR-3 (ovarian cancer), MDA-MD-231 (triple-negative breast cancer), SCC-1 (oral squamous carcinoma), HCT-116 (colon carcinoma), and MIA PaCa-2 (pancreatic carcinoma) [32,40,41]. Conversely, continuous NTX treatment has been associated with the increased proliferation (ranging from 9 to 71%) of various human and murine cancer types, with this effect claimed to be attributable to sustained opioid receptor blockades [22,32,42,43,44,45,46,47].

To the best of our knowledge, only two studies so far have directly investigated the effect of NTX in OSCC, both from the same research group. These studies have observed a 26% reduction in tumor cell proliferation compared to control with short-term NTX (72 h), while continuous 72 h treatment resulted in their increased proliferation [23,32]. Possible explanations for the discrepancy in our results may be attributed to the different cell lines utilized in the studies. Although all classified as OSCC, these cell lines are biologically and genetically different. For example, SCC-25 (used in our study) and SCC-1 (used by Donahue et al. [32] and Zagon et al. [23]) are both tongue squamous carcinoma cell lines, but differ in p53 status, OGFr expression, and OGF-OGFr axis activity, which could influence their response to NTX [48,49]. Similarly, Detroit 562 originates from pharyngeal carcinoma and manifests as more aggressive and less differentiated. Cell culture conditions, media supplements, drug stability, and dosing precision could have also influenced the different responses to NTX treatment.

### 3.2. NTX Effect Differs Between OSCC and Other Cell Types

In the current study, SKOV-3 was used as a positive control, given its consistent results across multiple studies. In SKOV-3 ovarian cells, intermittent NTX exposure modestly suppressed cell growth, consistent with prior reports [32,40,50]. Continuous NTX exposure resulted in mild growth stimulation at lower concentrations but was toxic at higher dosages, confirming an expected dose-dependent response and known off-target toxicity at elevated NTX concentrations [51]. Yet, opposing findings were observed by modulating cell density. These results suggest that NTX effects may be influenced by both cell type and density.

### 3.3. Inconsistent Effect of NTX in Combination with Chemotherapeutic Agents

Chemotherapeutic agents, such as cisplatin and docetaxel, are commonly used in the treatment of OSCC, especially for the locally advanced disease or in the advanced stages with regional or widespread metastasis. Nevertheless, their use is limited by numerous side effects, including nephrotoxicity, peripheral neuropathy, nausea, vomiting, and ototoxicity [52]. Such side effects are dose-dependent; thus, the exploration of combination therapies that allow for a reduction in the dosage—and therefore the toxicity—of chemotherapeutic agents is encouraged [53]. In our study, the leftward and downward shifts in the cell viability curves under NTX conditions, relative to controls without the NTX treatment, suggest that 10 µM NTX may potentiate the cytotoxic efficacy of both cisplatin and docetaxel in SCC-25 and Detroit 562 cells. Short-term NTX exposure (5 h and 24 h) generally resulted in lower cell viability compared to chemotherapy alone or the continuous NTX regimen, with the exception of the Detroit 562 treated with low-dose cisplatin (0–1.25 µM). Interestingly, at higher concentrations of cisplatin (5–20 µM), cisplatin alone produced greater cytotoxicity than when combined with either the 5 h or continuous NTX exposure. Our findings are consistent with similar in vitro studies conducted with regard to lung, colorectal carcinoma, and ovarian cancer, where the NTX regimen—such as single 48 h or repeated 6 h NTX treatment every other day over 5 days—resulted in a 20–45% tumor growth reduction in viability compared to the chemotherapy regimen alone (i.e., cyclophosphamide, gemcitabine, taxol, cisplatin, and oxaliplatin) [40,54]. However, full dose response and synergy analyses (Figure 3 and Figure 4) show that NTX and chemotherapy are largely antagonistic, regardless of the timing of treatment. Further studies are necessary in order to determine the differences underlying the discrepancies between these data and what has been previously reported. Such studies will contribute to the body of combination therapies that enhance tumor cell growth inhibition while allowing for lower doses of chemotherapeutic agents [55].

### 3.4. OGFr Expression and Modulation by NTX

In addition to its known binding capacity to μ-, δ-, and κ-opioid, NTX can also bind to the OGFr by competitively inhibiting the binding of the OGF. The OGF-OGFr complex has been reported to modulate cell proliferation by upregulating cyclin-dependent kinase inhibitors p16 and p21, resulting in the decreased phosphorylation of retinoblastoma protein (Rb) and the subsequent delay of the G1-to-S phase transition in the cycle phase [25,26,29]. Previous work has suggested that intermittent or low-dose NTX enhances OGF-OGFr signaling via a compensatory rebound mechanism, thereby inhibiting tumor growth, whereas continuous NTX exposure may suppress the OGFr expression and promote cell growth [21]. Our results suggested that OFGr is expressed in both SCC-25 and Detroit 562 cell lines, with a slightly higher baseline expression in Detroit 562 compared to normal controls. Various research has examined the OGF-OGFr axis across different cancer types, with some studies supporting the dysregulation of the pathway, especially in more aggressive and less differentiated tumors, and others reporting that the axis is maintained as intact or is only minimally altered [27,56,57]. Thus, alterations of the OGF-OGFr axis appear to be tumor-type-specific and potentially influenced by cancer stages [56,58]. 

We hypothesized that NTX would modulate the OGF-OGFr axis, and in turn, cell proliferation. By modulating this pathway, NTX would sensitize cells to the DNA-damaging effects of chemotherapeutic agents, resulting in a synergistic cytotoxicity, cell apoptosis, suppression of the cell cycle, and the promotion of an immunogenic microenvironment at lower chemotherapeutic doses [40,57,59]. Interestingly, our results showed that continuous NTX exposure resulted in a modest increase in the OFGr expression in SCC-25 cells, with inconsistent effects in Detroit 562 cells. Neither cell line showed a statistically significant change upon NTX treatment. Similarly, we did not observe a consistent upregulation in the OGFr expression under intermittent conditions. These findings contrast with the previous report that sustained opioid receptor blockade downregulates the OGFr [32] and that intermittent NTX exposure enhances OGF-OGFr signaling and suppresses proliferation. Such discrepancies may reflect tumor-type specificity, disease stage (i.e., advanced stage cells, such as Detroit 562, showing higher basal levels of OGFrs than earlier stage SCC-25 cells), or variability in the OGF-OGFr axis across cancer types [56]. Our results do not replicate the proposed pattern of NTX-induced OGF-OGFr modulation but may instead explain the lack of effect of NTX on the proliferation and survival in our experiments. Further work may be needed to optimize NTX dosing before an effect on the OGFr can be observed. As correlative changes in the OGFr expression alone cannot establish causality, future experiments should also incorporate loss-of-function or knockout and add-back approaches, or the assessment of downstream signaling markers, such as p16 or p21, to further support or refute a direct OGF-OGFr-mediated mechanistic effect. Collectively, our data suggest that the relationship between NTX exposure and OGF-OGFr signaling in OSCC may be more complex and context-dependent than previously described, necessitating further rigorous mechanistic validation.

### 3.5. Limitations

The current study has several limitations. As all experiments were performed in cell lines, not all factors present in human patients were accounted for. For example, no paracrine or microenvironment effects on tumor growth were accounted for in our models. In addition, statistical significance was impeded by the variability of our results, and further studies will be needed to identify the source(s) of this variation. Furthermore, due to the number of dosing schemes and drug combinations tested, treatment durations of 48–72 h were used for all experiments. In future, NTX treatments between 5 and 24 h may be tried, with overall assay durations greater than the 48–72 h used here. Additional studies will be necessary to determine the mechanism of NTX action on OSCC cells. OGFr levels were not significantly changed, especially in Detroit 562 cells, but the activity of the receptor and its downstream signals may still be impacted, as outlined above. Future studies with further optimized treatment protocols should focus on the signals downstream from OGFr. We also acknowledge that the presence of different factors in the media of the two cell lines, particularly hydrocortisone for SCC-25, may influence the OGF signals and NTX response in unforeseen ways. Finally, Donohue et al. observed that NTX exerted its effect in vivo in part via the inhibition of tumor angiogenesis [29]. No cell culture model can capture all factors present in vivo, and thus our approach cannot account for any effects NTX may have on the tumor microenvironment.

## 4. Materials and Methods

### 4.1. Chemicals, Drugs, and Reagents

Naltrexone hydrochloride and Sulphorhodamine B (SRB) were purchased from MilliporeSigma (Burlington, MA, USA). NTX was dissolved in water at a stock concentration of 10 mM and further diluted in growth media to the indicated final concentrations for each assay. Cisplatin was purchased from Avantor (Radnor, PA, USA). Docetaxel and Cell Counting Kit 8 (CCK-8) reagents were acquired from Selleck Chemicals (Houston, TX, USA). The additional CCK-8 reagent was purchased from GlpBio (Montclair, CA, USA). Hydrocortisone for growth media supplementation was purchased from STEMCELL Technologies (Cambridge, MA, USA).

### 4.2. Cell Lines and Cell Culture

In order to study two different stages of OSCC, two oropharyngeal carcinoma lines were selected. SCC-25 was derived from a locally invasive squamous cell carcinoma on the tongue of a 70-year-old male patient [33]. These cells were grown in DMEM-F12 media supplemented with 400 ng/mL hydrocortisone, 10% fetal bovine serum (FBS), and 1% penicillin-streptomycin (P/S). Detroit 562 was derived from a pleural effusion of a female patient with metastatic pharyngeal carcinoma, and therefore represents a more advanced disease [33]. These cells were maintained in EMEM supplemented with 10% FBS and 1% P/S. Normal primary gingival keratinocytes (PGK) were grown in Dermal basal media supplemented with a Keratinocyte Growth Kit from the American Type Culture Collection (ATCC, Manassas, VA, USA) and 1% P/S. SKOV-3 ovarian carcinoma cells were selected as positive controls for the effects of naltrexone, on the basis of prior studies [23,32,34]. SKOV-3 were grown in RPMI plus 10% FBS and 1% P/S. SKOV-3 cells were acquired from ATCC. PGK, SCC-25, and Detroit 562 cells were provided by Dr. Hilal Arnouk at Midwestern University. Cell cultures were maintained in a 37 °C, humidified incubator with 5% CO_2_ atmosphere, and were used within 12 passages of thaw to prevent drift.

### 4.3. Sulphorhodamine B Assays

The effects of NTX as a single agent were evaluated using an SRB assay. Briefly, cells were plated at 1500–3000 cells per well in 96-well plates. The following day, media was removed and replaced by NTX at 1 µM or 10 µM in normal media, or plain media as a control. NTX was added and removed, as outlined in Table 1, yielding treatments designated at 5 h once, 5 h daily, 5 h every other day (EOD), and constant exposure. Time points and doses were selected based on prior studies by Liubchenko et al. [21]. After 72 h, media and any remaining NTX were removed, and the cells were fixed in 100 µL per well 10% trichloroacetic acid (TCA) at 4 °C for one hour. TCA was then removed and the cells were washed with 200 µL water and allowed to air dry. The fixed cells were then stained with 0.4% SRB in 1% acetic acid for 15 min at room temperature. The SRB stain was discarded and wells were rinsed 3–4 times with 1% acetic acid until no further pink color was present in the discarded wash. The stray SRB around the walls of the wells was removed with a fine-point cotton swab, taking care not to disturb the stained cells on the bottom of the wells. SRB was solubilized in 200 µL per well 10 mM Tris base, pH 10.5, and plates were read for absorbance at 570 nm in an EnSpire Plate reader (Perkin Elmer, Waltham, MA, USA) to quantify SRB signal. The raw data were analyzed using Microsoft Excel, with normalizing to untreated control wells. A representative Excel analysis is given in the Supplementary Materials. All experiments were repeated at least three times with 4–5 technical replicates in each run.

### 4.4. Cell Counting Kit 8 Assays

Efficacy of combination therapy was evaluated via the Cell Counting Kit 8 (CCK-8) assay. Briefly, cells were plated at 3000 cells per well in 96-well plates. The following day, cells were treated with 10 µM NTX following the dosing regimens in Table 2, or with plain media control in a 100 µL total volume. Wells with media only and no cells were used for background subtraction. The following day, cisplatin or docetaxel was added in an additional 100 µL, bringing the total well volume to 200 µL. Cells were incubated for 48 h, and then 20 µL per well of CCK-8 reagent was added, including to the background wells. After a further 2 h incubation, plates were read at 450 nm in a BioTek Synergy H1 plate reader (Agilent Technologies, Santa Clara, CA, USA).

CCK-8 was also used for synergy determination experiments. Briefly, cells were plated at 3000 cells per well in 96-well plates. The following day, NTX was added to the cells. For co-treatment experiments, cisplatin or docetaxel was added at the same time in a total volume of 100 µL. Plates were then incubated for three days, after which 10 µL CCK-8 reagent was added and the incubation and data acquisition were conducted as described above. For the pretreatment experiments, NTX was removed after 5 h and replaced with normal medium. After 19 h, cisplatin or docetaxel was added in a total volume of 100 µL, and the plates were incubated for a further 48 h. CCK-8 assays were then performed as in the co-treatment condition. The synergy experimental scheme is shown in Table 3. Raw data were analyzed using Microsoft Excel, subtracting the average reading from the background wells from all the sample values and normalizing to untreated control wells. Representative Excel analyses for each experiment type are given in the Supplementary Materials. All experiments were repeated at least three times with three technical replicates per run.

### 4.5. Western Blotting

Cells with and without the NTX treatment were harvested using trypsin and were pelleted. Cell pellets were lysed in RIPA buffer supplemented with protease and phosphatase inhibitors and PMSF. Protein in lysates was quantified using BCA assay (ThermoFisher Scientific, Waltham, MA, USA), and the equal masses and volumes of protein were run on TGX precast gradient gels from Bio-Rad (Hercules, CA, USA). Protein was then transferred to PVDF membranes using the Transblot SD semi-dry transfer system from Bio-Rad. Membranes were blocked in 5% milk in phosphate-buffered saline with 0.5% Tween 20 (PBST) for 40 min at room temperature before incubation with primary antibodies in 5% bovine serum albumin in PBST overnight at 4 °C. Blots were then washed in PBST, incubated with secondary antibodies in 5% milk in PBST for 1 h at room temperature, washed again, and imaged using Clarity ECL substrate and a ChemiDoc imager, both from Bio-Rad. The band intensities were quantified using Bio-Rad Image Lab software, version 5.2.1. The primary antibodies were anti-OGFr (1:1000, #11177-1-AP from Proteintech, Rosemont, IL, USA) and anti-beta actin (1:500, #sc-47778 from Santa Cruz Biotechnology, Dallas, TX, USA). The secondary antibodies for OGFr and actin, respectively, were horse anti-rabbit (1:5000, #7074) and anti mouse (1:10,000, #7076) from Cell Signaling Technology (Danvers, MA, USA).

### 4.6. Statistics and Replication 

All assays were repeated at least three times unless otherwise noted. One-way ANOVA was used for single drug studies, and two-way ANOVA was used to analyze drug combination data. Drug interactions were evaluated using SynergyFinder 3.0 (https://synergyfinder.fimm.fi, accessed on 22 October 2025) [60].

## 5. Conclusions

We analyzed the effects of low-dose NTX on growth, response to chemotherapy, and OGFr expression in oropharyngeal carcinoma cell lines. In contrast to prior studies showing that intermittent dosing reduced cell growth while constant exposure increased it, we found that NTX did not reduce cell proliferation or survival, independent of dosing schedule. Select doses of NTX may improve responses to low doses of cisplatin or docetaxel, but, on the whole, NTX shows antagonism with chemotherapy under the conditions tested. Previous studies used different cell lines with different genetic characteristics, which may explain the discrepancy in our findings. Furthermore, in vitro studies such as ours cannot recapitulate prior encouraging in vivo results that emphasized altered tumor angiogenesis as a mechanism of action. Therefore, the effects of NTX on cancer cells appear highly context-dependent, and further study is warranted.

## Figures and Tables

**Table 1:** Treatment schemes for NTX single-agent experiments. NTX: naltrexone; SRB: Sulphorhodamine B. Constant: 72 h of continuous exposure; 5 h Once: one 5 h exposure; 5 h Daily: 5 h treatment daily for three days; and EOD: 5 h treatment every other day. Blue, drug added; red, drug removed. +, step performed; −, step not performed.

	Day 1	Day 2		Day 3		Day 4		Day 5
		NTX On	NTX Off	NTX On	NTX Off	NTX On	NTX Off	
Control	Plate cells	−	−	−	−	−	−	SRB Assay
Constant		+	−	+	−	+	−	
5 h Once		+	+	−	−	−	−	
5 h Daily		+	+	+	+	+	+	
5 h EOD		+	+	−	−	+	+	

**Table 2:** Treatment schemes for drug combination experiments. CCK-8: Cell Counting Kit 8; Chemo: chemotherapeutic agents (cisplatin or docetaxel); NTX: naltrexone. 5 h: 5 h pretreatment followed by 19 h normal media; 24 h: 24 h pretreatment; and Constant: continuous treatment (24 h pretreatment + continued NTX treatment during chemotherapy exposure). Blue, drug added; red, drug removed. +, step performed; −, step not performed.

	Day 1	Day 2		Day 3		Day 4	Day 5
		NTX On	NTX Off	NTX Off	Chemo On		
Control	Plate cells	−	−	−	+	Incubate	CCK-8 Assay
Constant		+	−	−	+		
5 h		+	+	−	+		
24 h		+	−	+	+		

**Table 3:** Treatment schemes for drug synergy experiments. CCK-8: Cell Counting Kit 8; Chemo: chemotherapeutic agents (cisplatin or docetaxel); and NTX: naltrexone. Blue, drug added; red, drug removed. +, step performed; −, step not performed.

	Day 1	Day 2			Day 3	Day 4	Day 5
		NTX On	NTX Off	Chemo On	Chemo On		
Co-treatment	Plate cells	+	−	+	−	Incubate	CCK-8 Assay
Pretreatment		+	+	−	+		

## Data Availability

The original contributions presented in this study are included in the article and Supplementary Materials. Further inquiries can be directed to the corresponding authors.

## Supplementary Materials

The following supporting information can be downloaded at: https://www.mdpi.com/article/10.3390/ijms262110651/s1.

## Abbreviations

The following abbreviations are used in this manuscript:

ANOVA	Analysis of variance
ATCC	American Type Culture Collection
CCK-8	Cell Counting Kit 8
CI	Confidence Interval
Cis	Cisplatin
Const	Constant treatment
Doce	Docetaxel
EOD	Every other day treatment
FBS	Fetal Bovine Serum
FDA	Food and Drug Administration
NT	No treatment
NTX	Naltrexone
OGF	Opioid growth factor
OGFr	Opioid growth factor receptor
OSCC	Oral squamous cell carcinoma
P/S	Penicillin-streptomycin
PBST	Phosphate-buffered saline + Tween20
PGK	Primary gingival keratinocytes
SD	Standard deviation
SRB	Sulphorhodamine B
TCA	Trichloroacetic acid
