# Supplementary material for: Naltrexone Has Variable and Schedule-Dependent Effects on Oral Squamous Cell Carcinoma Cells
Source: Int J Mol Sci. 2025 Nov 1;26(21):10651. doi: 10.3390/ijms262110651 (PMC12609663; doi:10.3390/ijms262110651)
Supplement: Supplementary file 1 [file ijms-26-10651-s001.zip › ijms-3858304-NTX in OSCC SUPP REVISION.pdf]

**Supplementary Information for:**

# Naltrexone Has Variable and Schedule-Dependent Effects on Oral Squamous Cell Carcinoma Cells

**Sahar Kazmi [1,†], Erica Sanford [1,†], Zaid A. Rammaha [2], Ethan J. Bengson [2], Feng Gao [1], Linda Sangalli [1,*] and Cai M. Roberts [3,*]**

[1] College of Dental Medicine—Illinois, Midwestern University, Downers Grove, IL 60515, USA; sahar.kazmi@midwestern.edu (S.K.); erica.sanford@midwestern.edu (E.S.); fgao@midwestern.edu (F.G.)
[2] Biomedical Sciences Program, Midwestern University, Downers Grove, IL 60515, USA; zaid.a.ramaha@gmail.com (Z.A.R.); ethan.bengson@midwestern.edu (E.J.B.)
[3] Department of Pharmacology, Midwestern University, Downers Grove, IL 60515, USA
* Correspondence: lsanga@midwestern.edu (L.S.); crober@midwestern.edu (C.M.R.)
† These authors contributed equally to this work, and each may be considered first author.

**Supplementary Figure S1**

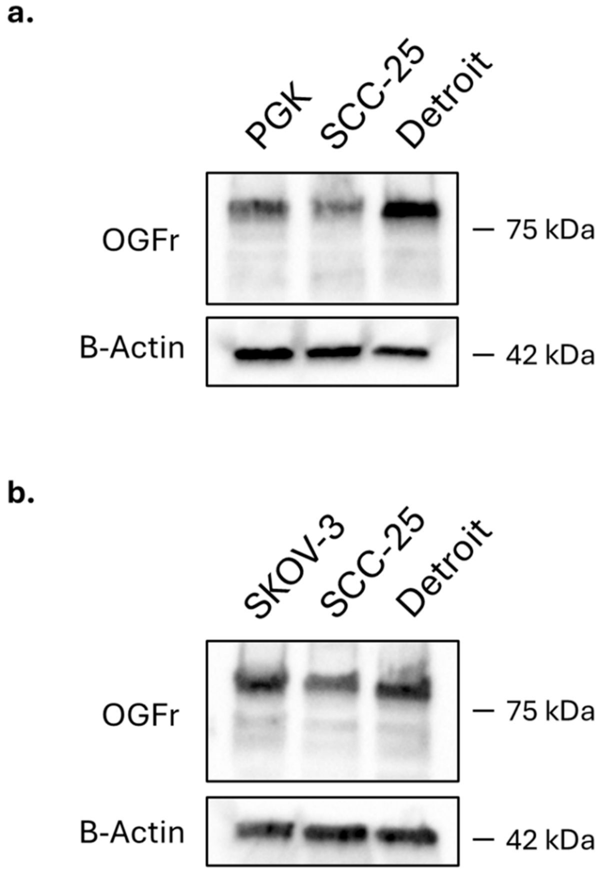


**Supplementary Figure S1.** (**a**) Western blot showing relative expression of OGFr in OSCC cell lines and in normal control cells (PGK). Detroit 562 shows the highest expression. (**b**) Western blot confirming similar levels of OGFr in SKOV-3 ovarian cancer cells as in OSCC lines. n=1 for each.

## Supplementary Figure S2

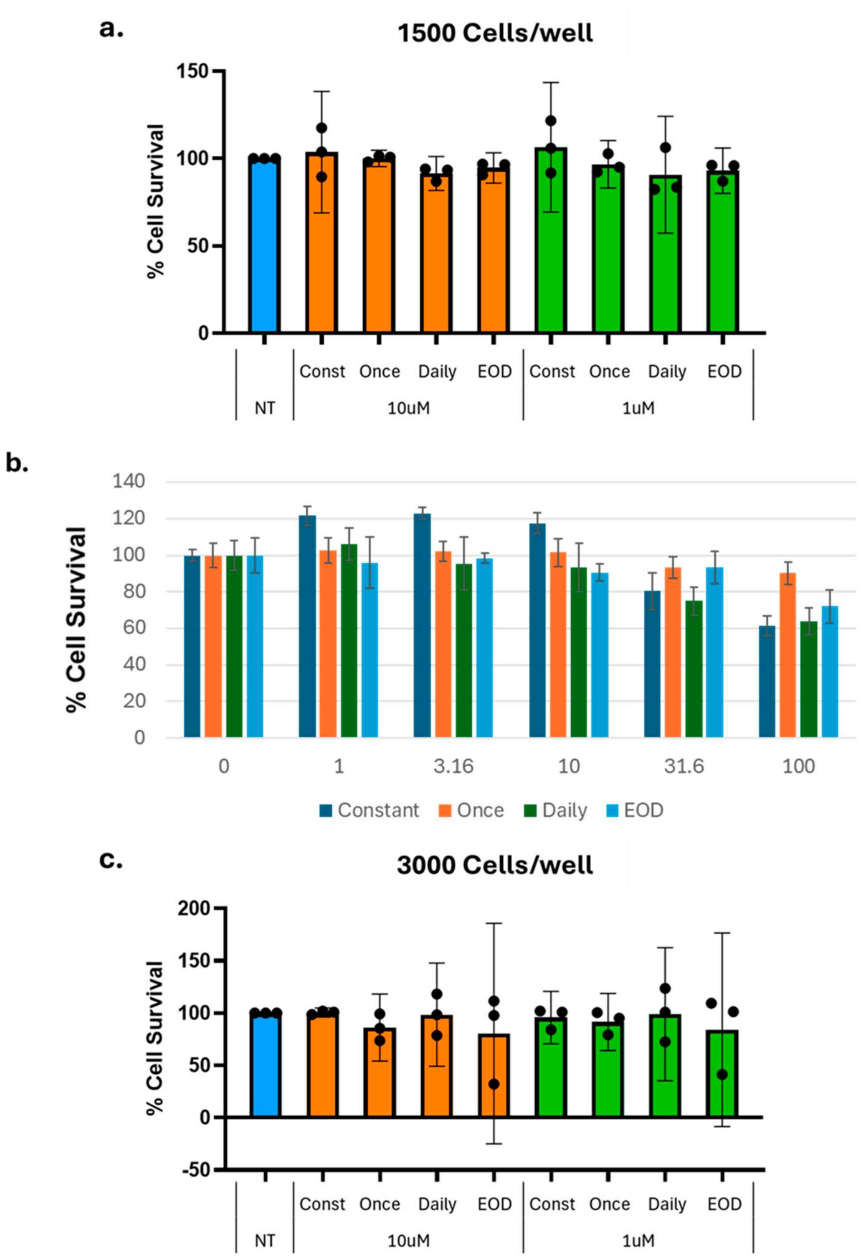


**Supplementary Figure S2.** Data from SKOV-3. (**a**) At 1500 cells/well, NTX shows little effect. Mean ± 95% CI shown. (**b**) Single experiment with expanded dose range shows NTX toxicity in SKOV-3 cells at high doses independent of dosing schedule. Graph shows mean ± SD of technical replicates. X-axis, dose of NTX in μM. **c.** Repeating the experiment in a. with 3000 cells/well shows a trend toward reduction in growth with NTX, but with high variability. Mean ± 95% CI shown. n=5 technical replicates except for a single experiment in a. with n=4 for select conditions (see complete raw data in Supplementary Information).

**Supplementary Figure S3**

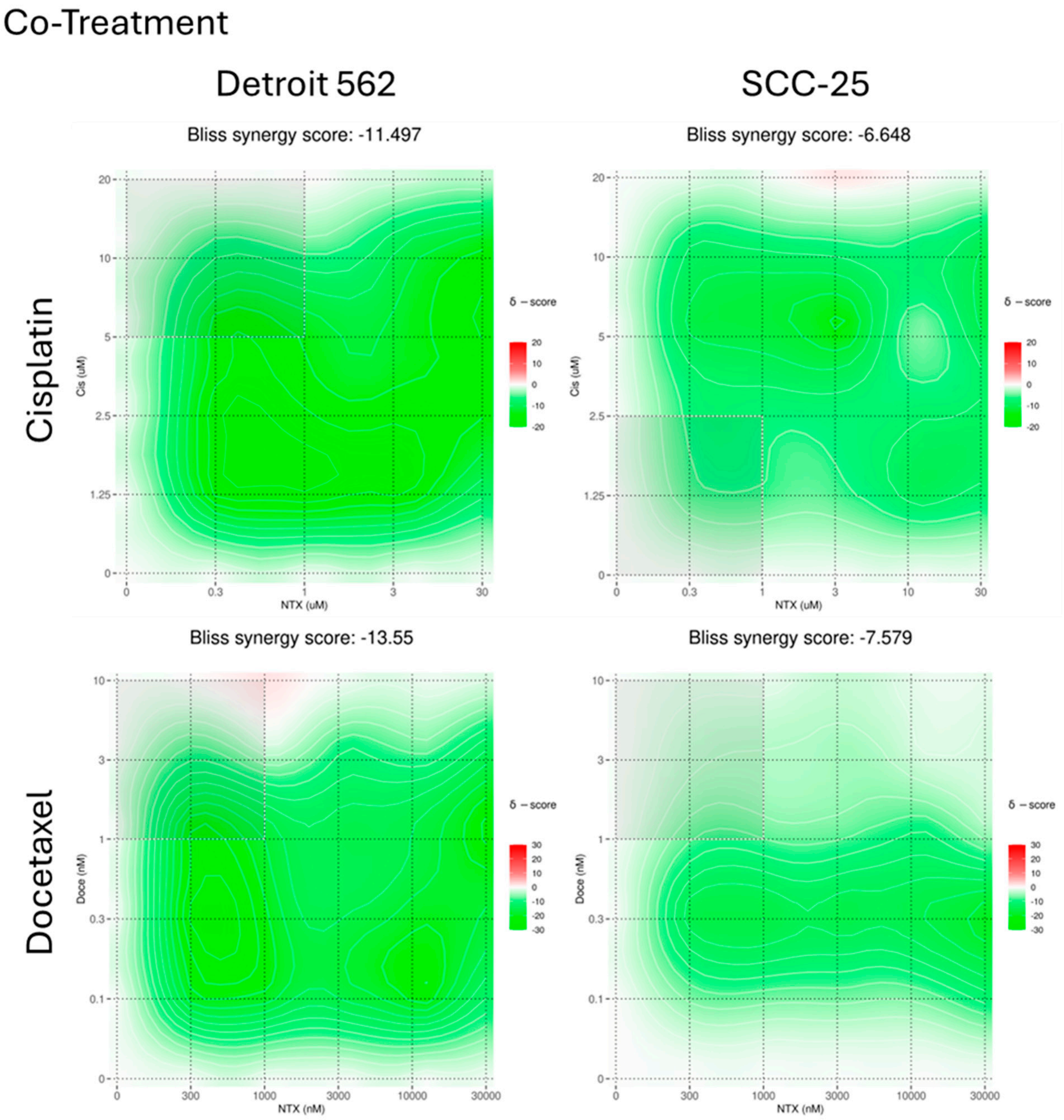


**Supplementary Figure S3.** Representative synergy plots for the indicated cell lines and drugs in combination with NTX co-treatment.

**Supplementary Figure S4**

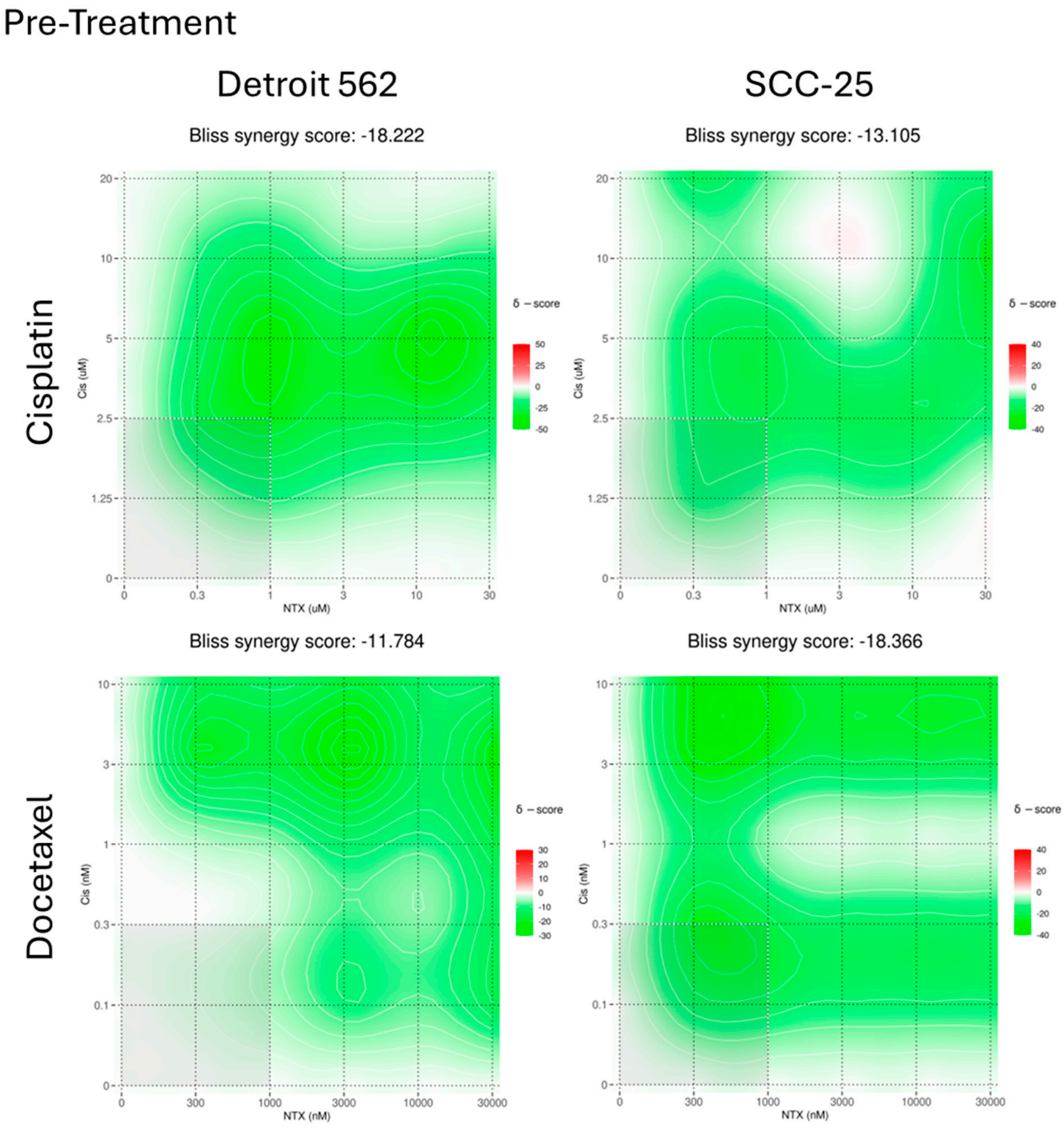


**Supplementary Figure S4.** Representative synergy plots for the indicated cell lines and drugs in combination with NTX pretreatment.

## Raw Data

### SRB Assays – Figure 1

Detroit 562

| | | | | | | | | | |
|---|---|---|---|---|---|---|---|---|---|
| 1.591 | 1.894 | 1.867 | 1.868 | 1.847 | 1.909 | 1.951 | 1.805 | 1.805 | 1.498 |
| 1.897 | 1.77 | 1.767 | 1.755 | 1.57 | 1.496 | 1.567 | 1.529 | 1.393 | 1.71 |
| 1.397 | 1.424 | 1.494 | 1.613 | 1.678 | 1.434 | 1.443 | 1.367 | 1.337 | 1.392 |
| 1.331 | 1.311 | 1.254 | 1.328 | 1.377 | 1.542 | 1.385 | 1.423 | 1.453 | 1.767 |
| 1.177 | 1.371 | 1.364 | 1.452 | 1.563 | 1.791 | 1.607 | 1.675 | 1.513 | 1.288 |

| | | | | | | | | | |
|---|---|---|---|---|---|---|---|---|---|
| 2.293 | 2.135 | 2.232 | 1.928 | 2.138 | 2.218 | 2.202 | 1.915 | 1.816 | 1.986 |
| 1.981 | 2.233 | 2.105 | 1.906 | 1.963 | 2.222 | 2.088 | 1.875 | 2.11 | 1.84 |
| 2.141 | 1.987 | 2.086 | 1.967 | 2.023 | 1.901 | 1.801 | 1.971 | 1.944 | 1.752 |
| 2.294 | 2.201 | 2.13 | 1.933 | 1.695 | 1.612 | 1.703 | 1.777 | 1.727 | 1.641 |
| 2.082 | 2.171 | 1.992 | 2.248 | 1.638 | 1.76 | 1.799 | 1.908 | 1.867 | 1.605 |

| | | | | | | | | | | |
|---|---|---|---|---|---|---|---|---|---|---|
| 0.61 | 0.88 | 0.816 | 0.828 | 0.851 | 0.876 | 0.831 | 0.943 | 0.812 | 0.665 | * |
| 0.571 | 0.948 | 0.82 | 0.686 | 0.691 | 0.743 | 0.93 | 0.721 | 0.762 | 0.42 | |
| 0.62 | 0.885 | 0.699 | 0.737 | 0.66 | 0.766 | 0.889 | 0.849 | 0.758 | 0.709 | |
| 1.038 | 0.891 | 0.917 | 0.805 | 0.909 | 0.696 | 0.791 | 0.883 | 0.87 | 0.862 | |
| 0.521 | 0.679 | 0.665 | 0.569 | 0.537 | 0.803 | 0.683 | 0.648 | 0.673 | 0.685 | |
| 0.676 | 0.753 | 0.771 | 0.648 | 0.575 | 0.73 | 0.797 | 0.683 | 0.599 | 0.662 | |

SCC-25

| | | | | | | | | | |
|---|---|---|---|---|---|---|---|---|---|
| 1.377 | 1.517 | 1.545 | 1.72 | 1.677 | 1.41 | 1.491 | 1.437 | 1.501 | 1.314 |
| 1.557 | 1.661 | 1.858 | 1.745 | 2.079 | 1.315 | 1.313 | 1.132 | 1.473 | 1.446 |
| 1.44 | 1.709 | 1.806 | 1.614 | 1.613 | 1.728 | 1.491 | 1.226 | 1.2 | 1.28 |
| 1.654 | 1.671 | 1.605 | 1.783 | 1.694 | 1.317 | 0.809 | 0.753 | 0.994 | 1.164 |
| 1.469 | 1.637 | 1.664 | 1.715 | 1.754 | 2.519 | 2.388 | 2.413 | 2.336 | 1.946 |

| | | | | | | | | | |
|---|---|---|---|---|---|---|---|---|---|
| 0.899 | 1.599 | 1.501 | 0.828 | 0.909 | 0.78 | 0.763 | 0.84 | 0.895 | 1.055 |
| 0.581 | 1.362 | 1.405 | 0.713 | 1.149 | 1.102 | 1.347 | 0.716 | 0.79 | 1.854 |
| 0.784 | 1.571 | 0.757 | 0.752 | 1.122 | 1.098 | 0.658 | 0.985 | 0.824 | 0.814 |
| 0.694 | 1.187 | 0.869 | 0.909 | 0.708 | 0.547 | 0.675 | 0.547 | 0.611 | 1.178 |
| 1.21 | 0.737 | 0.716 | 0.653 | 0.683 | 0.63 | 0.648 | 0.859 | 1.3 | 0.607 |

| | | | | | | | | | | |
|---|---|---|---|---|---|---|---|---|---|---|
| 0.571 | 0.948 | 0.82 | 0.686 | 0.691 | 0.743 | 0.93 | 0.721 | 0.762 | 0.42 | * |
| 0.514 | 0.615 | 0.565 | 0.552 | 0.595 | 0.542 | 0.644 | 0.484 | 0.471 | 0.36 | |
| 0.803 | 0.683 | 0.648 | 0.673 | 0.685 | 0.521 | 0.679 | 0.665 | 0.569 | 0.537 | |
| 0.73 | 0.797 | 0.683 | 0.599 | 0.662 | 0.676 | 0.753 | 0.771 | 0.648 | 0.575 | |
| 0.564 | 0.655 | 0.645 | 0.69 | 0.659 | 0.594 | 0.744 | 0.72 | 0.632 | 0.62 | |
| 0.635 | 0.642 | 0.654 | 0.59 | 0.52 | 0.662 | 0.65 | 0.72 | 0.664 | 0.539 | |

* Boxed values are all NT; extra NT wells included in this repeat.

# Drug Combination Assays – Figure 2

**Cisplatin**

Detroit 562

| | | | | | | | | | | | |
|---|---|---|---|---|---|---|---|---|---|---|---|
| 1.519 | 1.966 | 1.924 | 1.476 | 1.549 | 1.226 | 1.186 | 1.547 | 1.345 | 1.378 | 1.751 | 1.444 |
| 1.415 | 0.83 | 2.066 | 1.355 | 1.753 | 1.631 | 0.945 | 1.394 | 1.662 | 0.932 | 1.641 | 1.455 |
| 1.425 | 1.405 | 1.523 | 1.243 | 1.496 | 1.214 | 1.292 | 1.403 | 1.387 | 1.009 | 1.874 | 1.39 |
| 0.881 | 1.064 | 1.021 | 0.969 | 0.99 | 1.093 | 1.055 | 1.21 | 1.072 | 0.878 | 0.942 | 0.857 |
| 0.532 | 0.573 | 0.534 | 0.652 | 0.551 | 0.621 | 0.628 | 0.72 | 0.628 | 0.541 | 0.561 | 0.513 |
| 0.567 | 0.607 | 0.557 | 0.534 | 0.453 | 0.465 | 0.457 | 0.481 | 0.446 | 0.475 | 0.524 | 0.395 |
| | | | 0.225 | 0.218 | 0.214 | 0.219 | 0.219 | 0.225 | | | |

| | | | | | | | | | | | |
|---|---|---|---|---|---|---|---|---|---|---|---|
| 1.554 | 1.881 | 2.16 | 1.828 | 1.721 | 1.88 | 1.77 | 1.642 | 1.606 | 1.673 | 1.365 | 1.107 |
| 1.626 | 1.759 | 1.779 | 1.24 | 1.429 | 1.156 | 0.939 | 0.997 | 0.952 | 1.538 | 1.316 | 0.804 |
| 1.543 | 1.892 | 1.445 | 1.372 | 1.597 | 1.086 | 0.849 | 1.288 | 0.917 | 1.063 | 0.923 | 0.826 |
| 1.58 | 1.632 | 1.111 | 1.115 | 1.083 | 1.09 | 1.006 | 1.221 | 0.998 | 1.107 | 0.679 | 0.779 |
| 0.961 | 0.976 | 0.845 | 0.719 | 0.801 | 0.782 | 0.716 | 0.836 | 0.763 | 0.679 | 0.523 | 0.463 |
| 0.625 | 0.604 | 0.6 | 0.502 | 0.533 | 0.516 | 0.474 | 0.744 | 0.525 | 0.456 | 0.467 | 0.444 |
| | | | 0.297 | 0.296 | 0.306 | 0.347 | 0.3 | 0.294 | | | |

| | | | | | | | | | | | |
|---|---|---|---|---|---|---|---|---|---|---|---|
| 0.89 | 1.034 | 0.89 | 0.528 | 0.823 | 0.816 | 0.751 | 0.816 | 0.832 | 0.939 | 1.07 | 0.949 |
| 0.921 | 0.995 | 0.687 | 0.79 | 0.781 | 0.797 | 0.645 | 0.734 | 0.724 | 0.798 | 0.881 | 0.794 |
| 0.861 | 0.693 | 0.685 | 0.729 | 0.781 | 0.713 | 0.57 | 0.732 | 0.706 | 0.749 | 0.73 | 0.8 |
| 0.694 | 0.599 | 0.56 | 0.595 | 0.697 | 0.61 | 0.579 | 0.598 | 0.634 | 0.644 | 0.62 | 0.624 |
| 0.466 | 0.404 | 0.403 | 0.41 | 0.414 | 0.422 | 0.412 | 0.425 | 0.411 | 0.415 | 0.398 | 0.424 |
| 0.411 | 0.406 | 0.388 | 0.387 | 0.389 | 0.393 | 0.355 | 0.37 | 0.372 | 0.411 | 0.38 | 0.419 |
| | | | 0.224 | 0.233 | 0.238 | 0.236 | 0.237 | 0.229 | | | |

SCC-25

| | | | | | | | | | | | |
|---|---|---|---|---|---|---|---|---|---|---|---|
| 0.746 | 1.073 | 0.814 | 0.744 | 0.685 | 0.73 | 0.88 | 0.692 | 0.774 | 0.96 | 0.972 | 0.91 |
| 1.021 | 1.016 | 0.84 | 0.674 | 0.629 | 0.547 | 0.941 | 0.658 | 0.778 | 0.68 | 0.709 | 0.797 |
| 0.679 | 0.803 | 0.763 | 0.885 | 0.832 | 0.71 | 0.884 | 0.779 | 0.658 | 0.769 | 0.527 | 0.739 |
| 0.589 | 0.62 | 0.641 | 0.513 | 0.668 | 0.625 | 0.531 | 0.631 | 0.649 | 0.451 | 0.555 | 0.7 |
| 0.383 | 0.478 | 0.464 | 0.466 | 0.466 | 0.425 | 0.505 | 0.482 | 0.445 | 0.462 | 0.429 | 0.454 |
| 0.301 | 0.288 | 0.303 | 0.305 | 0.301 | 0.312 | 0.304 | 0.308 | 0.307 | 0.297 | 0.294 | 0.29 |
| | | | 0.216 | 0.207 | 0.213 | 0.216 | 0.212 | 0.215 | | | |

| | | | | | | | | | | | |
|---|---|---|---|---|---|---|---|---|---|---|---|
| 1.125 | 1.013 | 1.245 | 0.871 | 1.065 | 0.954 | 0.908 | 1.406 | 0.867 | 1.427 | 1.257 | 0.626 |
| 1.145 | 1.296 | 0.946 | 0.807 | 1.025 | 0.877 | 0.79 | 0.852 | 0.933 | 0.932 | 0.679 | 0.917 |
| 1.11 | 0.879 | 0.883 | 0.946 | 0.925 | 0.852 | 0.897 | 0.777 | 0.7 | 0.669 | 0.464 | 1.284 |
| 0.86 | 0.834 | 0.826 | 0.761 | 0.784 | 0.733 | 0.822 | 0.697 | 0.814 | 0.547 | 0.49 | 1.003 |
| 0.636 | 0.723 | 0.625 | 0.711 | 0.689 | 0.724 | 0.686 | 0.587 | 0.634 | 0.458 | 0.367 | 0.594 |
| 0.333 | 0.342 | 0.344 | 0.359 | 0.354 | 0.342 | 0.331 | 0.312 | 0.344 | 0.309 | 0.304 | 0.324 |
| | | | 0.28 | 0.281 | 0.282 | 0.29 | 0.29 | 0.295 | | | |

| | | | | | | | | | | | |
|---|---|---|---|---|---|---|---|---|---|---|---|
| 0.682 | 1.159 | 1.426 | 1.403 | 1.334 | 1.168 | 1.084 | 1.195 | 1.107 | 1.407 | 0.802 | 0.646 |
| 1.359 | 1.55 | 0.953 | 1.099 | 1.113 | 1.11 | 1.101 | 0.872 | 1.044 | 0.848 | 0.747 | 0.786 |
| 1.29 | 1.262 | 0.982 | 1.199 | 0.884 | 0.955 | 0.974 | 0.908 | 1.044 | 0.742 | 0.562 | 0.59 |
| 0.454 | 0.936 | 1.005 | 0.979 | 0.898 | 0.796 | 0.883 | 0.83 | 0.922 | 0.653 | 0.516 | 0.536 |
| 0.366 | 0.595 | 0.878 | 0.732 | 0.765 | 0.703 | 0.732 | 0.8 | 0.745 | 0.468 | 0.408 | 0.38 |
| 0.289 | 0.339 | 0.335 | 0.334 | 0.349 | 0.328 | 0.347 | 0.363 | 0.343 | 0.318 | 0.296 | 0.288 |
| | | | 0.216 | 0.222 | 0.224 | 0.23 | 0.225 | 0.227 | | | |

**Docetaxel**

Detroit 562

| | | | | | | | | | | | |
|---|---|---|---|---|---|---|---|---|---|---|---|
| 1.495 | 1.882 | 1.622 | 1.555 | 1.863 | 1.468 | 1.373 | 1.183 | 1.32 | 1.42 | 1.515 | 1.44 |
| 1.601 | 2.004 | 1.589 | 2.044 | 1.999 | 1.525 | 1.393 | 1.336 | 1.451 | 1.594 | 1.236 | 1.567 |
| 1.773 | 1.845 | 1.961 | 1.611 | 1.535 | 1.924 | 1.431 | 1.338 | 1.378 | 1.872 | 1.742 | 1.726 |
| 1.44 | 1.601 | 1.441 | 1.633 | 1.643 | 1.418 | 1.284 | 1.285 | 1.232 | 1.394 | 1.308 | 1.448 |
| 1.013 | 1.102 | 1.05 | 1.156 | 0.99 | 1.016 | 0.863 | 0.917 | 0.84 | 0.899 | 0.92 | 1.067 |
| 0.737 | 0.69 | 0.697 | 0.722 | 0.734 | 0.716 | 0.677 | 0.624 | 0.655 | 0.736 | 0.685 | 0.712 |
| | | | 0.316 | 0.311 | 0.312 | 0.306 | 0.305 | 0.297 | | | |

| | | | | | | | | | | | |
|---|---|---|---|---|---|---|---|---|---|---|---|
| 1.434 | 1.523 | 1.485 | 1.513 | 1.442 | 1.395 | 1.276 | 1.301 | 1.314 | 1.436 | 0.95 | 1.104 |
| 1.404 | 1.409 | 1.209 | 1.17 | 1.055 | 1.106 | 0.97 | 1.1 | 1.136 | 1.199 | 0.98 | 0.991 |
| 1.387 | 1.273 | 1.339 | 1.334 | 1.083 | 1.049 | 0.976 | 1.227 | 1.078 | 1.027 | 0.912 | 0.774 |
| 1.206 | 1.072 | 1.071 | 1.255 | 0.929 | 0.961 | 0.896 | 0.982 | 0.956 | 0.905 | 0.759 | 0.814 |
| 0.774 | 0.837 | 0.751 | 0.837 | 0.69 | 0.625 | 0.699 | 0.734 | 0.789 | 0.6 | 0.554 | 0.587 |
| 0.578 | 0.572 | 0.587 | 0.621 | 0.532 | 0.556 | 0.555 | 0.497 | 0.621 | 0.546 | 0.465 | 0.491 |
| | | | 0.283 | 0.29 | 0.293 | 0.282 | 0.291 | 0.293 | | | |

| | | | | | | | | | | | |
|---|---|---|---|---|---|---|---|---|---|---|---|
| 2.682 | 2.755 | 2.512 | 2.321 | 2.649 | 2.373 | 2.266 | 2.429 | 1.957 | 2.521 | 2.511 | 2.584 |
| 2.639 | 2.758 | 2.613 | 2.604 | 2.543 | 2.226 | 2.386 | 2.523 | 2.466 | 2.604 | 2.567 | 2.411 |
| 2.558 | 2.693 | 2.532 | 2.544 | 2.658 | 2.378 | 2.375 | 2.32 | 2.035 | 2.274 | 2.385 | 2.373 |
| 2.258 | 2.374 | 2.206 | 2.047 | 2.257 | 2.083 | 1.768 | 2.162 | 1.922 | 1.869 | 1.988 | 2.013 |
| 2.003 | 1.95 | 1.632 | 1.505 | 1.579 | 1.913 | 1.28 | 1.234 | 1.443 | 1.292 | 1.707 | 1.672 |
| 0.97 | 0.949 | 0.861 | 0.885 | 0.85 | 0.749 | 0.838 | 0.872 | 0.681 | 0.832 | 0.924 | 0.876 |
| | | | 0.263 | 0.26 | 0.264 | 0.243 | 0.245 | 0.249 | | | |

| | | | | | | | | | | | |
|---|---|---|---|---|---|---|---|---|---|---|---|
| 1.477 | 1.907 | 1.981 | 1.839 | 1.487 | 1.919 | 1.644 | 1.872 | 1.489 | 1.789 | 1.865 | 1.687 |
| 1.665 | 1.828 | 1.405 | 1.834 | 1.479 | 1.532 | 1.263 | 1.556 | 1.455 | 1.594 | 1.601 | 1.859 |
| 1.662 | 1.886 | 1.622 | 1.685 | 1.684 | 1.602 | 1.374 | 1.351 | 1.148 | 1.253 | 1.471 | 1.68 |
| 1.45 | 1.662 | 1.449 | 1.453 | 1.399 | 1.374 | 1.191 | 1.242 | 1.119 | 1.253 | 1.377 | 1.495 |
| 1.015 | 1.187 | 1.042 | 1.065 | 1.116 | 1.085 | 0.888 | 0.764 | 0.819 | 1.031 | 0.98 | 1.028 |
| 0.614 | 0.717 | 0.733 | 0.703 | 0.727 | 0.68 | 0.612 | 0.607 | 0.573 | 0.653 | 0.681 | 0.723 |
| | | | 0.276 | 0.275 | 0.274 | 0.272 | 0.275 | 0.271 | | | |

SCC-25

| | | | | | | | | | | | |
|---|---|---|---|---|---|---|---|---|---|---|---|
| 1.475 | 1.223 | 1.338 | 1.06 | 1.175 | 1.197 | 0.928 | 0.803 | 1.07 | 1.365 | 1.308 | 1.706 |
| 1.396 | 1.727 | 1.065 | 1.122 | 1.324 | 1.248 | 1.231 | 0.775 | 0.714 | 1.094 | 1.112 | 1.319 |
| 1.467 | 1.264 | 1.288 | 0.985 | 1.167 | 1.145 | 1.056 | 1.117 | 0.926 | 1.083 | 0.976 | 1.101 |
| 1.197 | 0.914 | 1.041 | 0.814 | 0.917 | 0.776 | 0.92 | 0.884 | 0.64 | 0.761 | 0.881 | 0.848 |
| 0.856 | 0.793 | 0.815 | 0.657 | 0.792 | 0.753 | 0.782 | 0.674 | 0.541 | 0.663 | 0.65 | 0.66 |
| 0.61 | 0.574 | 0.597 | 0.5 | 0.567 | 0.566 | 0.561 | 0.533 | 0.453 | 0.525 | 0.507 | 0.522 |
| | | | 0.285 | 0.286 | 0.281 | 0.282 | 0.285 | 0.283 | | | |

| | | | | | | | | | | | |
|---|---|---|---|---|---|---|---|---|---|---|---|
| 0.721 | 1.041 | 1.152 | 1.165 | 0.918 | 1.021 | 0.735 | 0.967 | 0.869 | 1.016 | 0.944 | 0.831 |
| 1.03 | 0.941 | 0.882 | 1.047 | 0.845 | 0.768 | 0.702 | 0.772 | 0.81 | 0.815 | 0.769 | 0.796 |
| 0.876 | 0.965 | 0.636 | 0.773 | 0.737 | 0.778 | 0.703 | 0.619 | 0.543 | 0.612 | 0.656 | 0.667 |
| 0.703 | 0.64 | 0.365 | 0.53 | 0.557 | 0.501 | 0.459 | 0.47 | 0.455 | 0.504 | 0.506 | 0.527 |
| 0.556 | 0.49 | 0.375 | 0.453 | 0.447 | 0.436 | 0.359 | 0.452 | 0.391 | 0.405 | 0.454 | 0.453 |
| 0.393 | 0.415 | 0.35 | 0.381 | 0.393 | 0.397 | 0.341 | 0.402 | 0.381 | 0.358 | 0.38 | 0.393 |
| | | | 0.273 | 0.267 | 0.266 | 0.277 | 0.275 | 0.27 | | | |

| | | | | | | | | | | | |
|---|---|---|---|---|---|---|---|---|---|---|---|
| 1.349 | 1.375 | 1.449 | 1.296 | 0.841 | 0.802 | 0.768 | 0.724 | 0.796 | 1.171 | 1.233 | 1.289 |
| 1.198 | 1.278 | 1.238 | 0.825 | 0.725 | 0.706 | 0.718 | 0.761 | 0.799 | 0.655 | 0.924 | 1.47 |
| 1.238 | 0.873 | 1.068 | 0.853 | 0.736 | 0.727 | 0.78 | 0.738 | 0.82 | 0.603 | 0.872 | 1.471 |
| 0.867 | 1.1 | 0.836 | 0.662 | 0.655 | 0.657 | 0.597 | 0.571 | 0.588 | 0.439 | 0.516 | 1.305 |
| 0.841 | 0.57 | 0.647 | 0.558 | 0.548 | 0.544 | 0.504 | 0.441 | 0.485 | 0.445 | 0.42 | 0.502 |
| 0.63 | 0.473 | 0.446 | 0.488 | 0.455 | 0.438 | 0.409 | 0.361 | 0.428 | 0.402 | 0.392 | 0.355 |
| | | | 0.246 | 0.251 | 0.248 | 0.251 | 0.25 | 0.256 | | | |

| | | | | | | | | | | | |
|---|---|---|---|---|---|---|---|---|---|---|---|
| 1.034 | 0.963 | 0.967 | 0.901 | 1.024 | 0.958 | 0.906 | 0.924 | 0.923 | 0.925 | 0.915 | 1.038 |
| 0.952 | 0.936 | 0.946 | 0.976 | 0.98 | 0.972 | 0.887 | 0.946 | 0.956 | 0.92 | 0.949 | 0.923 |
| 0.851 | 0.827 | 0.872 | 0.9 | 0.869 | 0.836 | 0.816 | 0.798 | 0.855 | 0.912 | 0.858 | 0.883 |
| 0.565 | 0.525 | 0.57 | 0.578 | 0.59 | 0.569 | 0.532 | 0.55 | 0.591 | 0.545 | 0.577 | 0.636 |
| 0.492 | 0.429 | 0.483 | 0.476 | 0.465 | 0.448 | 0.452 | 0.449 | 0.468 | 0.48 | 0.477 | 0.518 |
| 0.362 | 0.346 | 0.357 | 0.354 | 0.378 | 0.373 | 0.382 | 0.364 | 0.378 | 0.368 | 0.366 | 0.394 |
| | | | 0.243 | 0.246 | 0.244 | 0.237 | 0.24 | 0.24 | | | |

## Drug Synergy Assays – Figures 3 and 4

**Co-treatment**

Detroit 562 + Cisplatin

| | | | | | | | | | | | |
|---|---|---|---|---|---|---|---|---|---|---|---|
| 1.737 | 2.972 | 2.526 | 2.173 | 2.378 | 1.713 | 1.989 | 2.006 | 2.26 | 1.951 | 2.066 | 1.524 |
| 1.442 | 1.904 | 2.25 | 2.239 | 2.262 | 2.33 | 2.261 | 2.232 | 2.196 | 2.036 | 1.97 | 1.736 |
| 1.178 | 1.697 | 1.892 | 1.887 | 1.808 | 1.822 | 1.697 | 1.852 | 1.753 | 1.767 | 1.755 | 1.188 |
| 0.712 | 1.018 | 1.318 | 1.26 | 1.249 | 1.21 | 1.386 | 1.163 | 1.251 | 1.293 | 1.187 | 1.041 |
| 0.331 | 0.387 | 0.469 | 0.493 | 0.491 | 0.483 | 0.477 | 0.544 | 0.519 | 0.5 | 0.52 | 0.514 |
| 0.206 | 0.227 | 0.241 | 0.217 | 0.212 | 0.209 | 0.212 | 0.221 | 0.202 | 0.204 | 0.199 | 0.219 |
| | | | 0.152 | 0.157 | 0.155 | 0.155 | 0.156 | 0.16 | | | |

| | | | | | | | | | | | |
|---|---|---|---|---|---|---|---|---|---|---|---|
| 1.129 | 1.522 | 1.371 | 1.417 | 1.425 | 2.951 | 1.493 | 1.475 | 1.513 | 1.526 | 1.55 | 1.201 |
| 1.096 | 1.286 | 1.375 | 1.407 | 1.432 | 1.484 | 1.395 | 1.435 | 1.487 | 1.504 | 1.196 | 1.287 |
| 1.046 | 1.415 | 1.508 | 1.498 | 1.502 | 1.506 | 1.429 | 1.446 | 1.54 | 1.664 | 1.586 | 1.144 |
| 1.02 | 1.51 | 1.472 | 1.595 | 1.619 | 1.448 | 1.536 | 1.395 | 1.736 | 1.864 | 1.844 | 1.522 |
| 0.576 | 0.949 | 0.827 | 0.833 | 0.79 | 0.777 | 0.94 | 0.856 | 0.856 | 0.845 | 0.896 | 0.941 |
| 0.301 | 0.315 | 0.303 | 0.262 | 0.264 | 0.29 | 0.277 | 0.324 | 0.288 | 0.336 | 0.334 | 0.365 |
| | | | 0.14 | 0.146 | 0.135 | 0.136 | 0.14 | 0.142 | | | |

| | | | | | | | | | | | |
|---|---|---|---|---|---|---|---|---|---|---|---|
| 1.426 | 1.636 | 1.65 | 1.695 | 1.418 | 1.59 | 1.53 | 1.712 | 1.737 | 1.517 | 1.564 | 1.596 |
| 1.43 | 1.651 | 1.713 | 1.789 | 1.856 | 1.959 | 2.02 | 1.858 | 1.802 | 1.694 | 1.846 | 1.539 |
| 1.426 | 1.822 | 1.741 | 2.091 | 2.108 | 2.082 | 2.084 | 2.141 | 2.006 | 2.146 | 2.154 | 1.869 |
| 1.344 | 1.351 | 1.597 | 1.669 | 1.396 | 1.63 | 1.338 | 1.685 | 1.522 | 1.557 | 1.535 | 1.386 |
| 0.515 | 0.723 | 0.769 | 0.827 | 0.69 | 0.636 | 0.676 | 0.709 | 0.711 | 0.708 | 0.671 | 0.535 |
| 0.198 | 0.22 | 0.214 | 0.217 | 0.2 | 0.242 | 0.243 | 0.229 | 0.22 | 0.235 | 0.289 | 0.221 |
| | | | 0.147 | 0.15 | 0.155 | 0.156 | 0.152 | 0.159 | | | |

Detroit 562 + Docetaxel

| | | | | | | | | | | | |
|---|---|---|---|---|---|---|---|---|---|---|---|
| 1.81 | 2.794 | 1.858 | 2.072 | 1.936 | 1.953 | 1.974 | 1.938 | 2.14 | 2.24 | 2.371 | 1.36 |
| 1.299 | 1.836 | 2.672 | 2.69 | 2.738 | 3.071 | 3.125 | 2.816 | 2.918 | 2.963 | 2.962 | 2.534 |
| 1.278 | 1.615 | 2.744 | 2.698 | 2.627 | 2.684 | 2.996 | 2.827 | 2.945 | 2.87 | 2.869 | 2.003 |
| 1.428 | 2.188 | 2.628 | 2.711 | 2.729 | 2.758 | 2.721 | 2.776 | 2.786 | 2.961 | 2.693 | 2.303 |
| 0.948 | 0.955 | 1.173 | 0.997 | 0.945 | 1.039 | 1.053 | 1.074 | 1.044 | 1.053 | 0.95 | 1.085 |
| 0.395 | 0.563 | 0.641 | 0.528 | 0.553 | 0.539 | 0.601 | 0.557 | 0.504 | 0.53 | 0.585 | 0.5 |
| | | | 0.168 | 0.163 | 0.14 | 0.144 | 0.149 | 0.141 | | | |

| | | | | | | | | | | | |
|---|---|---|---|---|---|---|---|---|---|---|---|
| 1.129 | 1.522 | 1.371 | 1.417 | 1.425 | 2.951 | 1.493 | 1.475 | 1.513 | 1.526 | 1.55 | 1.201 |
| 1.096 | 1.286 | 1.375 | 1.407 | 1.432 | 1.484 | 1.395 | 1.435 | 1.487 | 1.504 | 1.196 | 1.287 |
| 1.046 | 1.415 | 1.508 | 1.498 | 1.502 | 1.506 | 1.429 | 1.446 | 1.54 | 1.664 | 1.586 | 1.144 |
| 1.02 | 1.51 | 1.472 | 1.595 | 1.619 | 1.448 | 1.536 | 1.395 | 1.736 | 1.864 | 1.844 | 1.522 |
| 0.576 | 0.949 | 0.827 | 0.833 | 0.79 | 0.777 | 0.94 | 0.856 | 0.856 | 0.845 | 0.896 | 0.941 |
| 0.301 | 0.315 | 0.303 | 0.262 | 0.264 | 0.29 | 0.277 | 0.324 | 0.288 | 0.336 | 0.334 | 0.365 |
| | | | 0.14 | 0.146 | 0.135 | 0.136 | 0.14 | 0.142 | | | |

| | | | | | | | | | | | |
|---|---|---|---|---|---|---|---|---|---|---|---|
| 1.06 | 1.354 | 1.443 | 1.426 | 1.564 | 1.566 | 1.707 | 1.552 | 1.519 | 1.532 | 1.558 | 1.277 |

| | | | | | | | | | | | |
|---|---|---|---|---|---|---|---|---|---|---|---|
| 1.112 | 1.428 | 1.521 | 1.649 | 1.584 | 1.696 | 1.6 | 1.723 | 1.667 | 1.62 | 1.673 | 1.396 |
| 1.075 | 1.447 | 1.535 | 1.585 | 1.633 | 1.617 | 1.679 | 1.618 | 1.712 | 1.702 | 1.745 | 1.275 |
| 1.068 | 1.528 | 1.726 | 1.723 | 1.683 | 1.627 | 1.681 | 1.626 | 1.59 | 1.664 | 1.718 | 1.359 |
| 1.281 | 1.738 | 1.944 | 2.07 | 1.883 | 1.994 | 1.903 | 1.911 | 1.614 | 2.098 | 1.98 | 1.452 |
| 0.593 | 0.731 | 0.842 | 0.879 | 0.838 | 0.779 | 0.737 | 0.857 | 0.788 | 0.867 | 0.886 | 0.653 |
| | | | 0.146 | 0.15 | 0.15 | 0.139 | 0.145 | 0.144 | | | |

SCC-25 + Cisplatin

| | | | | | | | | | | | |
|---|---|---|---|---|---|---|---|---|---|---|---|
| 1.239 | 1.684 | 1.545 | 1.535 | 1.566 | 1.419 | 1.533 | 1.458 | 1.339 | 1.446 | 1.244 | 1.603 |
| 1.24 | 1.498 | 1.422 | 1.515 | 1.52 | 1.496 | 1.541 | 1.548 | 1.41 | 1.528 | 1.585 | 1.524 |
| 1.237 | 1.473 | 1.449 | 1.528 | 1.427 | 1.498 | 1.429 | 1.552 | 1.353 | 1.461 | 1.557 | 1.436 |
| 1.149 | 1.345 | 1.374 | 1.407 | 1.391 | 1.402 | 1.721 | 1.348 | 1.256 | 1.245 | 1.372 | 1.402 |
| 0.728 | 0.824 | 0.888 | 0.882 | 0.839 | 0.907 | 0.418 | 1.3 | 0.83 | 0.854 | 0.911 | 0.903 |
| 0.24 | 0.273 | 0.247 | 0.255 | 0.254 | 0.245 | 0.231 | 0.219 | 0.225 | 0.236 | 0.242 | 0.278 |
| | | | 0.142 | 0.138 | 0.137 | 0.133 | 0.139 | 0.136 | | | |

| | | | | | | | | | | | |
|---|---|---|---|---|---|---|---|---|---|---|---|
| 1.053 | 1.325 | 1.453 | 1.452 | 1.527 | 1.278 | 1.395 | 1.25 | 1.434 | 1.224 | 1.216 | 1.211 |
| 1.029 | 1.609 | 1.617 | 1.616 | 1.583 | 1.655 | 1.692 | 1.743 | 1.754 | 1.677 | 1.253 | 1.385 |
| 1.133 | 1.361 | 1.464 | 1.707 | 1.688 | 1.698 | 1.519 | 1.64 | 1.81 | 1.567 | 1.548 | 1.211 |
| 0.975 | 1.54 | 1.617 | 1.756 | 1.696 | 1.457 | 1.673 | 1.409 | 1.767 | 1.47 | 1.458 | 1.248 |
| 0.822 | 1.169 | 1.363 | 1.3 | 1.411 | 1.424 | 1.367 | 1.185 | 1.192 | 1.359 | 1.226 | 1.041 |
| 0.641 | 0.952 | 0.747 | 0.822 | 0.915 | 0.971 | 0.83 | 0.814 | 0.849 | 0.882 | 0.812 | 0.825 |
| | | | 0.134 | 0.137 | 0.142 | 0.146 | 0.143 | 0.131 | | | |

| | | | | | | | | | | | |
|---|---|---|---|---|---|---|---|---|---|---|---|
| 1.312 | 1.497 | 1.394 | 1.349 | 1.365 | 1.487 | 1.397 | 1.311 | 1.471 | 1.449 | 1.5 | 1.367 |
| 1.022 | 1.309 | 1.204 | 1.122 | 1.068 | 1.18 | 1.124 | 1.029 | 1.237 | 1.196 | 1.304 | 1.045 |
| 0.954 | 1.085 | 1.108 | 1.092 | 1.329 | 1.036 | 1.015 | 1.009 | 1.077 | 0.925 | 0.935 | 0.902 |
| 0.588 | 0.756 | 0.75 | 0.691 | 0.741 | 0.714 | 0.677 | 0.69 | 0.62 | 0.608 | 0.666 | 0.639 |
| 0.266 | 0.34 | 0.271 | 0.304 | 0.332 | 0.289 | 0.281 | 0.31 | 0.275 | 0.259 | 0.294 | 0.218 |
| 0.159 | 0.178 | 0.16 | 0.167 | 0.177 | 0.174 | 0.171 | 0.16 | 0.179 | 0.16 | 0.161 | 0.157 |
| | | | 0.146 | 0.142 | 0.146 | 0.15 | 0.133 | 0.137 | | | |

SCC-25 + Docetaxel

| | | | | | | | | | | | |
|---|---|---|---|---|---|---|---|---|---|---|---|
| 1.123 | 1.628 | 1.674 | 1.651 | 1.724 | 1.709 | 1.692 | 1.653 | 1.648 | 1.425 | 1.64 | 1.36 |
| 1.133 | 1.536 | 1.663 | 1.769 | 1.705 | 1.795 | 1.748 | 1.845 | 1.736 | 1.634 | 1.879 | 1.274 |
| 1.078 | 1.413 | 1.647 | 1.653 | 1.688 | 1.796 | 1.767 | 1.656 | 1.76 | 1.791 | 1.802 | 1.323 |
| 1.03 | 1.384 | 1.622 | 1.671 | 1.688 | 1.771 | 1.747 | 1.676 | 1.785 | 1.711 | 1.769 | 1.365 |
| 1.081 | 1.157 | 1.642 | 1.577 | 1.411 | 1.772 | 1.794 | 1.697 | 1.806 | 1.778 | 1.841 | 1.341 |
| 1.054 | 1.239 | 1.465 | 1.572 | 1.571 | 1.653 | 1.701 | 1.649 | 1.776 | 1.733 | 1.8 | 1.227 |
| | | | 0.126 | 0.128 | 0.131 | 0.142 | 0.143 | 0.149 | | | |

| | | | | | | | | | | | |
|---|---|---|---|---|---|---|---|---|---|---|---|
| 1.398 | 1.51 | 1.5 | 1.57 | 1.541 | 1.514 | 1.5 | 1.5 | 1.432 | 1.416 | 1.52 | 1.518 |
| 1.368 | 1.482 | 1.564 | 1.592 | 1.585 | 1.543 | 1.539 | 1.453 | 1.516 | 1.485 | 1.419 | 1.457 |

| | | | | | | | | | | | |
|---|---|---|---|---|---|---|---|---|---|---|---|
| 1.565 | 1.565 | 1.616 | 1.548 | 1.638 | 1.587 | 1.548 | 1.471 | 1.533 | 1.468 | 1.478 | 1.526 |
| 1.335 | 1.378 | 1.401 | 1.532 | 1.444 | 1.495 | 1.299 | 1.309 | 1.389 | 1.351 | 1.339 | 1.313 |
| 0.547 | 0.625 | 0.665 | 0.658 | 0.604 | 0.721 | 0.673 | 0.65 | 0.658 | 0.608 | 0.625 | 0.59 |
| 0.433 | 0.497 | 0.505 | 0.518 | 0.42 | 0.454 | 0.463 | 0.404 | 0.426 | 0.451 | 0.433 | 0.419 |
| | | | 0.341 | 0.111 | 0.117 | 0.125 | 0.167 | 0.237 | | | |

| | | | | | | | | | | | |
|---|---|---|---|---|---|---|---|---|---|---|---|
| 1.213 | 1.487 | 1.482 | 1.527 | 1.46 | 1.563 | 1.478 | 1.452 | 1.413 | 1.498 | 1.282 | 1.43 |
| 1.197 | 1.475 | 1.524 | 1.526 | 1.514 | 1.573 | 1.528 | 1.481 | 1.486 | 1.572 | 1.6 | 1.508 |
| 1.08 | 1.358 | 1.442 | 1.472 | 1.462 | 1.517 | 1.477 | 1.507 | 1.441 | 1.448 | 1.507 | 1.398 |
| 0.631 | 0.79 | 0.806 | 0.8 | 0.793 | 0.802 | 0.812 | 0.787 | 0.797 | 0.889 | 0.778 | 0.647 |
| 0.412 | 0.483 | 0.486 | 0.497 | 0.568 | 0.43 | 0.489 | 0.532 | 0.469 | 0.464 | 0.465 | 0.471 |
| 0.31 | 0.358 | 0.34 | 0.361 | 0.374 | 0.3 | 0.34 | 0.4 | 0.321 | 0.371 | 0.307 | 0.369 |
| | | | 0.152 | 0.134 | 0.136 | 0.147 | 0.163 | 0.141 | | | |

**Pretreatment**

Detroit 562 + Cisplatin

| | | | | | | | | | | | |
|---|---|---|---|---|---|---|---|---|---|---|---|
| 0.923 | 1.17 | 1.381 | 1.629 | 1.371 | 1.638 | 1.353 | 1.65 | 1.087 | 1.454 | 1.432 | 0.695 |
| 1.363 | 1.232 | 1.327 | 1.438 | 1.642 | 2.066 | 1.613 | 2.084 | 1.507 | 1.77 | 1.238 | 1.202 |
| 1.111 | 1.268 | 1.353 | 1.602 | 1.767 | 2.091 | 1.872 | 2.016 | 2.038 | 2.09 | 1.409 | 1.513 |
| 1.599 | 1.51 | 1.527 | 1.669 | 1.751 | 2.053 | 2.116 | 1.931 | 2.235 | 2.675 | 1.657 | 1.583 |
| 1.203 | 1.154 | 1.121 | 0.967 | 1.274 | 1.136 | 1.433 | 1.796 | 1.555 | 2.243 | 1.233 | 1.168 |
| 0.418 | 0.468 | 0.471 | 0.494 | 0.44 | 0.388 | 0.523 | 0.572 | 0.558 | 0.858 | 0.543 | 0.519 |
| | | | 0.117 | 0.146 | 0.148 | 0.15 | 0.134 | 0.137 | | | |

| | | | | | | | | | | | |
|---|---|---|---|---|---|---|---|---|---|---|---|
| 1.844 | 1.955 | 2.257 | 2.47 | 2.072 | 2.172 | 2.083 | 2.876 | 2.943 | 2.665 | 2.195 | 2.033 |
| 1.664 | 2.449 | 2.257 | 2.23 | 2.504 | 2.099 | 2.614 | 2.299 | 2.257 | 2.178 | 2.318 | 1.868 |
| 1.53 | 1.778 | 2.607 | 2.249 | 2.175 | 2.745 | 2.291 | 2.429 | 2.64 | 2.652 | 2.289 | 1.728 |
| 1.09 | 1.446 | 1.62 | 1.844 | 1.895 | 2.15 | 2.193 | 1.496 | 2.299 | 2.126 | 1.879 | 1.644 |
| 0.823 | 1.027 | 1.187 | 1.284 | 1.313 | 1.279 | 1.025 | 1.224 | 1.234 | 1.012 | 1.11 | 1.205 |
| 0.352 | 0.449 | 0.497 | 0.447 | 0.548 | 0.407 | 0.504 | 0.45 | 0.436 | 0.447 | 0.428 | 0.465 |
| | | | 0.157 | 0.156 | 0.145 | 0.158 | 0.158 | 0.114 | | | |

| | | | | | | | | | | | |
|---|---|---|---|---|---|---|---|---|---|---|---|
| 1.141 | 1.509 | 1.487 | 1.4 | 1.538 | 2.082 | 1.76 | 1.751 | 1.592 | 1.879 | 1.692 | 1.81 |
| 1.318 | 1.549 | 2.146 | 1.913 | 1.712 | 1.76 | 1.893 | 1.917 | 1.721 | 2.555 | 2.461 | 1.535 |
| 1.63 | 1.314 | 2.234 | 2.302 | 2.432 | 2.339 | 2.037 | 2.344 | 2.515 | 2.469 | 2.493 | 1.573 |
| 1.415 | 1.763 | 2.134 | 2.297 | 2.125 | 2.176 | 2.229 | 2.19 | 2.142 | 2.095 | 2.119 | 1.229 |
| 0.888 | 1.167 | 1.349 | 1.38 | 1.363 | 1.393 | 1.4 | 1.315 | 1.54 | 1.283 | 1.224 | 0.774 |
| 0.371 | 0.444 | 0.645 | 0.586 | 0.619 | 0.614 | 0.586 | 0.614 | 0.595 | 0.494 | 0.395 | 0.361 |
| | | | 0.151 | 0.152 | 0.145 | 0.121 | 0.135 | 0.144 | | | |

Detroit 562 + Docetaxel

| | | | | | | | | | | | |
|---|---|---|---|---|---|---|---|---|---|---|---|
| 1.547 | 2.355 | 2.075 | 2.25 | 2.183 | 2.102 | 2.161 | 2.118 | 2.147 | 2.183 | 2.17 | 2.35 |
| 1.782 | 2.259 | 2.212 | 2.205 | 2.035 | 2.161 | 2.297 | 2.331 | 2.327 | 2.249 | 2.241 | 2.399 |
| 1.609 | 2.254 | 2.461 | 2.526 | 2.216 | 2.293 | 2.204 | 2.194 | 2.156 | 2.231 | 2.169 | 2.303 |
| 1.585 | 2.261 | 2.253 | 2.181 | 2.258 | 2.262 | 2.322 | 2.178 | 2.114 | 2.168 | 2.091 | 2.31 |
| 1.567 | 1.957 | 2.025 | 2.062 | 2.207 | 2.117 | 2.071 | 2.036 | 1.817 | 2.016 | 1.925 | 2.279 |
| 1.041 | 1.226 | 1.297 | 1.432 | 1.29 | 1.273 | 1.35 | 1.355 | 1.26 | 1.359 | 1.251 | 1.349 |
| | | | 0.15 | 0.148 | 0.154 | 0.148 | 0.153 | 0.152 | | | |

| | | | | | | | | | | | |
|---|---|---|---|---|---|---|---|---|---|---|---|
| 2.168 | 2.393 | 2.42 | 2.394 | 1.931 | 2.728 | 2.129 | 2.264 | 2.249 | 2.276 | 2.035 | 2.084 |
| 1.971 | 2.724 | 3.182 | 2.763 | 2.902 | 2.536 | 2.853 | 2.432 | 2.354 | 2.716 | 2.721 | 2.589 |
| 1.914 | 3.138 | 3.009 | 3.023 | 2.862 | 2.442 | 3.371 | 2.858 | 3.207 | 2.934 | 3.394 | 2.527 |
| 1.9 | 2.694 | 2.71 | 3.209 | 2.902 | 3.248 | 3.285 | 2.719 | 2.96 | 2.512 | 2.853 | 2.741 |
| 1.629 | 2.208 | 2.559 | 2.233 | 2.261 | 2.27 | 2.519 | 2.415 | 1.824 | 2.368 | 2.487 | 1.953 |
| 1.159 | 1.395 | 1.642 | 1.535 | 1.445 | 1.528 | 1.645 | 1.425 | 1.329 | 1.579 | 1.363 | 1.433 |
| | | | 0.161 | 0.159 | 0.159 | 0.157 | 0.154 | 0.157 | | | |

| | | | | | | | | | | | |
|---|---|---|---|---|---|---|---|---|---|---|---|
| 1.167 | 1.149 | 1.236 | 1.165 | 1.517 | 1.433 | 1.204 | 1.4 | 1.399 | 1.467 | 1.339 | 1.202 |
| 1.155 | 1.111 | 1.591 | 1.694 | 1.632 | 1.693 | 1.633 | 1.66 | 1.73 | 1.782 | 1.713 | 1.271 |
| 1.168 | 1.087 | 1.485 | 1.692 | 1.723 | 1.673 | 1.607 | 1.664 | 1.664 | 1.787 | 1.64 | 1.259 |
| 1.117 | 1.111 | 1.589 | 1.68 | 1.622 | 1.697 | 1.633 | 1.675 | 1.579 | 1.739 | 1.739 | 1.414 |
| 1.147 | 1.137 | 1.59 | 1.671 | 1.691 | 1.782 | 1.846 | 1.817 | 1.801 | 1.876 | 1.819 | 1.248 |
| 1.041 | 1.108 | 1.46 | 1.552 | 1.609 | 1.574 | 1.683 | 1.818 | 1.731 | 1.658 | 1.489 | 1.094 |
| | | | 0.136 | 0.147 | 0.143 | 0.145 | 0.149 | 0.147 | | | |

SCC-25 + Cisplatin

| | | | | | | | | | | | |
|---|---|---|---|---|---|---|---|---|---|---|---|
| 0.821 | 1.403 | 1.519 | 1.587 | 1.601 | 1.312 | 1.104 | 1.562 | 1.6 | 1.527 | 1.628 | 1.084 |
| 0.797 | 1.384 | 1.474 | 1.551 | 1.63 | 1.556 | 1.579 | 1.579 | 1.629 | 1.573 | 1.579 | 0.955 |
| 0.802 | 1.169 | 1.609 | 1.634 | 1.648 | 1.648 | 1.639 | 1.628 | 1.655 | 1.67 | 1.675 | 0.966 |
| 0.843 | 1.306 | 1.46 | 1.562 | 1.536 | 1.54 | 1.541 | 1.558 | 1.554 | 1.593 | 1.591 | 0.939 |
| 0.715 | 0.833 | 1.178 | 1.252 | 1.264 | 1.24 | 1.23 | 1.12 | 1.253 | 1.428 | 1.371 | 0.797 |
| 0.316 | 0.366 | 0.414 | 0.441 | 0.454 | 0.469 | 0.439 | 0.46 | 0.482 | 0.558 | 0.488 | 0.484 |
| | | | 0.131 | 0.131 | 0.132 | 0.129 | 0.133 | 0.131 | | | |

| | | | | | | | | | | | |
|---|---|---|---|---|---|---|---|---|---|---|---|
| 1.22 | 1.819 | 1.517 | 1.413 | 1.826 | 1.902 | 1.514 | 1.793 | 2.006 | 1.902 | 1.65 | 1.502 |
| 1.132 | 1.703 | 1.597 | 1.521 | 1.766 | 1.716 | 1.728 | 1.696 | 1.784 | 1.629 | 1.505 | 1.299 |
| 0.89 | 1.631 | 1.616 | 1.252 | 1.646 | 1.675 | 1.434 | 1.663 | 1.639 | 1.811 | 1.529 | 1.384 |
| 0.797 | 1.53 | 1.591 | 1.135 | 1.429 | 1.479 | 1.121 | 1.417 | 1.549 | 1.223 | 1.476 | 1.468 |
| 0.755 | 0.942 | 1.039 | 0.799 | 1.009 | 0.792 | 0.803 | 0.842 | 0.99 | 0.978 | 1.193 | 1.37 |
| 0.288 | 0.554 | 0.892 | 0.515 | 0.576 | 0.57 | 0.409 | 0.513 | 0.547 | 0.588 | 0.429 | 0.423 |
| | | | 0.143 | 0.142 | 0.141 | 0.142 | 0.136 | 0.141 | | | |

| | | | | | | | | | | | |
|---|---|---|---|---|---|---|---|---|---|---|---|
| 1.799 | 1.825 | 2.131 | 2.369 | 1.846 | 1.859 | 1.593 | 1.821 | 1.647 | 1.815 | 1.749 | 1.695 |
| 1.688 | 1.783 | 1.774 | 1.798 | 1.783 | 1.896 | 2.145 | 1.707 | 1.638 | 1.768 | 1.612 | 1.71 |
| 1.663 | 1.732 | 1.681 | 1.746 | 1.637 | 1.685 | 2.075 | 1.625 | 1.645 | 1.627 | 1.558 | 1.32 |

| | | | | | | | | | | | |
|---|---|---|---|---|---|---|---|---|---|---|---|
| 1.518 | 1.522 | 1.499 | 1.812 | 1.392 | 1.431 | 1.923 | 1.495 | 1.615 | 1.504 | 1.425 | 1.442 |
| 1.268 | 1.534 | 1.493 | 1.285 | 1.63 | 1.249 | 1.113 | 1.177 | 1.127 | 1.857 | 1.125 | 1.086 |
| 0.52 | 0.407 | 0.377 | 0.384 | 0.35 | 0.353 | 0.361 | 0.391 | 0.421 | 0.495 | 0.412 | 0.432 |
| | | | 0.158 | 0.16 | 0.158 | 0.158 | 0.156 | 0.159 | | | |

SCC-25 + Docetaxel

| | | | | | | | | | | | |
|---|---|---|---|---|---|---|---|---|---|---|---|
| 1.13 | 1.326 | 1.134 | 1.266 | 1.173 | 1.476 | 1.437 | 1.504 | 1.505 | 1.479 | 1.466 | 1.452 |
| 1.057 | 1.31 | 1.264 | 1.287 | 1.414 | 1.622 | 1.604 | 1.653 | 1.61 | 1.559 | 1.52 | 1.37 |
| 0.924 | 1.415 | 1.532 | 1.634 | 1.435 | 1.63 | 1.661 | 1.702 | 1.551 | 1.615 | 1.5 | 1.43 |
| 1.226 | 1.48 | 1.405 | 1.497 | 1.53 | 1.718 | 1.614 | 1.655 | 1.631 | 1.594 | 1.556 | 1.483 |
| 0.897 | 1.273 | 1.544 | 1.525 | 1.602 | 1.656 | 1.674 | 1.696 | 1.663 | 1.483 | 1.613 | 1.457 |
| 0.884 | 1.301 | 1.503 | 1.265 | 1.484 | 1.563 | 1.627 | 1.618 | 1.587 | 1.446 | 1.468 | 1.458 |
| | | | 0.137 | 0.138 | 0.14 | 0.139 | 0.139 | 0.137 | | | |

| | | | | | | | | | | | |
|---|---|---|---|---|---|---|---|---|---|---|---|
| 1.169 | 1.724 | 1.784 | 1.824 | 1.379 | 1.731 | 1.818 | 1.822 | 1.382 | 1.33 | 1.494 | 1.152 |
| 1.119 | 1.474 | 1.773 | 1.499 | 1.668 | 1.788 | 1.424 | 1.369 | 1.504 | 1.577 | 1.484 | 1.336 |
| 1.078 | 1.612 | 1.707 | 1.714 | 1.717 | 1.739 | 1.749 | 1.716 | 1.673 | 1.789 | 1.909 | 1.395 |
| 0.998 | 1.238 | 1.411 | 1.367 | 1.427 | 1.448 | 1.444 | 1.371 | 1.405 | 1.168 | 1.46 | 1.453 |
| 0.656 | 0.906 | 0.909 | 0.917 | 0.956 | 0.996 | 1.017 | 0.956 | 0.981 | 0.976 | 0.855 | 0.978 |
| 0.589 | 0.75 | 0.72 | 0.644 | 0.561 | 0.609 | 0.687 | 0.701 | 0.548 | 0.6 | 0.615 | 0.641 |
| | | | 0.151 | 0.152 | 0.15 | 0.151 | 0.139 | 0.154 | | | |

| | | | | | | | | | | | |
|---|---|---|---|---|---|---|---|---|---|---|---|
| 2.19 | 2.772 | 2.812 | 1.592 | 1.595 | 1.655 | 1.614 | 1.592 | 3.15 | 1.484 | 1.465 | 2.932 |
| 1.131 | 1.642 | 2.916 | 1.994 | 1.943 | 1.98 | 1.923 | 1.925 | 2.16 | 3.626 | 1.895 | 1.195 |
| 2.002 | 1.708 | 1.886 | 3.588 | 1.878 | 1.967 | 1.885 | 1.916 | 2.837 | 3.641 | 3.409 | 1.576 |
| 0.967 | 1.574 | 1.845 | 3.268 | 1.76 | 1.819 | 1.776 | 1.772 | 1.812 | 1.851 | 3.223 | 1.634 |
| 0.709 | 1.071 | 1.225 | 1.31 | 1.237 | 1.29 | 1.202 | 1.282 | 1.301 | 1.351 | 2.34 | 1.112 |
| 1.207 | 0.799 | 0.812 | 0.831 | 0.868 | 1.417 | 0.982 | 0.839 | 0.916 | 1.501 | 0.819 | 0.757 |
| | | | 0.169 | 0.166 | 0.15 | 0.148 | 0.15 | 0.144 | | | |

# SKOV -3 Data – Supplementary Figure S2

## 1500 cells/well

| | | | | | | | | | |
|---|---|---|---|---|---|---|---|---|---|
| 0.278 | 0.328 | 0.334 | 0.292 | 0.279 | 0.284 | 0.299 | 0.291 | 0.31 | 0.296 |
| 0.255 | 0.226 | 0.287 | 0.283 | 0.289 | 0.304 | 0.314 | 0.323 | 0.325 | 0.319 |
| 0.279 | 0.287 | 0.289 | 0.302 | 0.309 | 0.285 | 0.222 | 0.326 | 0.258 | 0.289 |
| 0.163 | 0.296 | 0.298 | 0.287 | 0.257 | 0.245 | 0.276 | 0.292 | 0.149 | 0.271 |
| 0.294 | 0.291 | 0.272 | 0.286 | 0.305 | 0.287 | 0.311 | 0.301 | 0.309 | 0.231 |

| | | | | | | | | | |
|---|---|---|---|---|---|---|---|---|---|
| 0.477 | 0.469 | 0.5 | 0.493 | 0.439 | 0.469 | 0.442 | 0.443 | 0.445 | 0.436 |
| 0.397 | 0.546 | 0.491 | 0.494 | 0.467 | 0.456 | 0.409 | 0.43 | 0.428 | 0.396 |
| 0.474 | 0.528 | 0.476 | 0.428 | 0.418 | 0.401 | 0.433 | 0.421 | 0.496 | 0.442 |
| 0.421 | 0.378 | 0.47 | 0.422 | 0.479 | 0.394 | 0.4 | 0.402 | 0.406 | 0.324 |
| 0.508 | 0.445 | 0.433 | 0.428 | 0.413 | 0.411 | 0.371 | 0.404 | 0.386 | 0.435 |

| | | | | | | | | | | |
|---|---|---|---|---|---|---|---|---|---|---|
| 0.227 | 0.234 | 0.241 | 0.223 | 0.224 | 0.215 | 0.234 | 0.23 | 0.258 | 0.243 | * |
| 0.161 | 0.148 | 0.164 | 0.18 | 0.173 | 0.194 | 0.206 | 0.181 | 0.218 | | |
| 0.259 | 0.292 | 0.263 | 0.271 | 0.266 | 0.289 | 0.28 | 0.294 | 0.27 | 0.265 | |
| 0.223 | 0.235 | 0.224 | 0.262 | 0.255 | 0.231 | 0.231 | 0.247 | 0.235 | 0.269 | |
| 0.144 | 0.151 | 0.132 | 0.183 | | 0.161 | 0.161 | 0.185 | 0.187 | | ** |
| 0.183 | 0.187 | 0.167 | 0.166 | 0.178 | 0.139 | 0.202 | 0.195 | 0.207 | 0.19 | |

* Boxed values are all NT; extra NT wells included in this repeat.

** n=4 for Daily, EOD conditions

## 3000 cells/well

| | | | | | | | | | |
|---|---|---|---|---|---|---|---|---|---|
| 0.648 | 0.562 | 0.509 | 0.546 | 0.522 | 0.577 | 0.581 | 0.595 | 0.539 | 0.558 |
| 0.528 | 0.535 | 0.541 | 0.582 | 0.593 | 0.588 | 0.584 | 0.578 | 0.57 | 0.556 |
| 0.556 | 0.564 | 0.555 | 0.573 | 0.552 | 0.584 | 0.586 | 0.552 | 0.552 | 0.558 |
| 0.543 | 0.572 | 0.572 | 0.558 | 0.532 | 0.559 | 0.603 | 0.582 | 0.557 | 0.542 |
| 0.57 | 0.546 | 0.566 | 0.55 | 0.525 | 0.587 | 0.562 | 0.564 | 0.568 | 0.578 |

| | | | | | | | | | |
|---|---|---|---|---|---|---|---|---|---|
| 1.125 | 1.032 | 0.95 | 0.983 | 1 | 0.942 | 0.995 | 0.936 | 0.996 | 0.924 |
| 1.04 | 1.081 | 0.961 | 0.923 | 0.98 | 0.869 | 0.849 | 0.741 | 0.79 | 0.905 |
| 0.772 | 0.892 | 0.859 | 0.865 | 0.839 | 0.839 | 0.831 | 0.79 | 0.638 | 0.821 |
| 0.767 | 0.834 | 0.697 | 0.691 | 0.894 | 0.666 | 0.708 | 0.688 | 0.748 | 0.775 |
| 0.203 | 0.294 | 0.285 | 0.438 | 0.366 | 0.431 | 0.605 | 0.455 | 0.278 | 0.275 |

| | | | | | | | | | | |
|---|---|---|---|---|---|---|---|---|---|---|
| 0.884 | 0.908 | 0.894 | 0.907 | 0.789 | 0.929 | 1.002 | 0.78 | 0.774 | 0.668 | * |
| 0.713 | 0.691 | 0.64 | 0.482 | 0.762 | 0.758 | 0.616 | 0.517 | 0.855 | 0.814 | |
| 0.847 | 0.822 | 1 | 0.759 | 1.043 | 0.9 | 0.718 | 0.804 | 0.94 | 1.058 | |
| 0.864 | 0.663 | 0.682 | 0.373 | 0.475 | 1.069 | 0.902 | 0.791 | 0.633 | 0.551 | |
| 0.702 | 0.773 | 0.8 | 0.79 | 0.823 | 0.874 | 0.818 | 0.826 | 0.733 | 0.816 | |
| 0.872 | 0.812 | 0.775 | 0.808 | 0.702 | 0.761 | 0.811 | 0.795 | 0.751 | 0.778 | |

* Boxed values are all NT; extra NT wells included in this repeat.
